# Joint Inversion of GNSS and GRACE for Terrestrial Water Storage Change in California

**DOI:** 10.1029/2021JB023135

**Published:** 2022-03-25

**Authors:** G. Carlson, S. Werth, M. Shirzaei

**Affiliations:** ^1^ Department of Geological Sciences Virginia Polytechnic and State University Blacksburg VA USA

**Keywords:** elastic loading, GNSS, GRACE, drought monitoring, California, terrestrial water storage change

## Abstract

Global Navigation Satellite System (GNSS) vertical displacements measuring the elastic response of Earth's crust to changes in hydrologic mass have been used to produce terrestrial water storage change (∆TWS) estimates for studying both annual ∆TWS as well as multi‐year trends. However, these estimates require a high observation station density and minimal contamination by nonhydrologic deformation sources. The Gravity Recovery and Climate Experiment (GRACE) is another satellite‐based measurement system that can be used to measure regional TWS fluctuations. The satellites provide highly accurate ∆TWS estimates with global coverage but have a low spatial resolution of ∼400 km. Here, we put forward the mathematical framework for a joint inversion of GNSS vertical displacement time series with GRACE ∆TWS to produce more accurate spatiotemporal maps of ∆TWS, accounting for the observation errors, data gaps, and nonhydrologic signals. We aim to utilize the regional sensitivity to ∆TWS provided by GRACE mascon solutions with higher spatial resolution provided by GNSS observations. Our approach utilizes a continuous wavelet transform to decompose signals into their building blocks and separately invert for long‐term and short‐term mass variations. This allows us to preserve trends, annual, interannual, and multi‐year changes in TWS that were previously challenging to capture by satellite‐based measurement systems or hydrological models, alone. We focus our study in California, USA, which has a dense GNSS network and where recurrent, intense droughts put pressure on freshwater supplies. We highlight the advantages of our joint inversion results for a tectonically active study region by comparing them against inversion results that use only GNSS vertical deformation as well as with maps of ∆TWS from hydrological models and other GRACE solutions. We find that our joint inversion framework results in a solution that is regionally consistent with the GRACE ∆TWS solutions at different temporal scales but has an increased spatial resolution that allows us to differentiate between regions of high and low mass change better than using GRACE alone.

## Key Points

- A new joint inversion framework combining Global Navigation Satellite System and Gravity Recovery and Climate Experiment (GRACE) is put forward to calculate monthly terrestrial water storage change (∆TWS) over California from 2003 to 2016
- A wavelet‐based approach is used to maintain spectral frequency content, allowing for preservation of multi‐ to sub‐annual signal components
- Inversion results show improved isolation of larger magnitude signals over regions where we expect large ∆TWS as compared to GRACE alone

## 1 Introduction

Accurately estimating the change in terrestrial water storage (∆TWS), which is the sum of the changes in groundwater, surface water, soil moisture, ice, and snow over the land surface, is important for monitoring the health and security of human populations and natural ecosystems. Negative TWS trends across the globe have been attributed to ice cap melting due to global warming (e.g., Hugonnet et al., 2021; Rignot et al., 2019; Velicogna et al., 2014), excessive anthropogenic groundwater withdrawals (e.g., Rodell et al., 2009; Wada et al., 2012), and drought (e.g., Griffin & Anchukaitis, 2014; van Dijk et al., 2013), among others (Rodell et al., 2018). If global climate trends continue, monitoring ∆TWS will become increasingly important as some regions are likely to experience increased wetness, potentially leading to higher flood risk, while other regions are likely to experience reduced snowpack and increased dryness leading to more intense drought (USGCRP, 2018). In arid and semiarid regions, future climatic and anthropogenic drivers of freshwater loss will put additional stress on the agricultural sector (USGCRP, 2018; WWAP, 2012). California, USA, which produces more than half of the United States' vegetables, fruits, and nuts (CDFA, 2020), has experienced a decline in TWS over the last two decades driven by repeated droughts and excessive groundwater withdrawals (e.g., Faunt et al., 2015). Monitoring ∆TWS in agricultural areas like California will be essential to maintaining regional food security.

∆TWS can only be measured directly at local scales (∼10^2^ m) through in situ point‐source observations that may have poor spatiotemporal coverage or predicted through hydrological water budget or water balance models (Güntner et al., 2007). However, hydrological models often fail to accurately represent long‐term changes or reflect the impact of human water abstraction on water storage (Argus et al., 2017; Scanlon et al., 2018). In addition, across much of the globe, the number of in situ measurements are limited and often contain temporal and spatial data gaps, especially for contributions from surface water (Solander et al., 2016) and groundwater (Frappart & Ramillien, 2018).

∆TWS can also be quantified using the interaction of the water cycle with the solid Earth and its gravity field. The Gravity Recovery and Climate Experiment (GRACE) and its follow‐on twin satellites, launched in 2002 and 2018, respectively, have allowed scientists to estimate regional ∆TWS with a sensitivity of nearly 1 cm equivalent water thickness across regions of ∼300–400 km with global coverage and monthly temporal resolution (Scanlon et al., 2012; Tapley et al., 2019). The ∆TWS measurements are inverted from spatiotemporal variations in Earth's gravity field found through precise measurements of distance and speed variations between the two satellites. Because GRACE observes the total water mass change over large areas, it has been essential in regional closure of the water budget (Rodell et al., 2015; Sheffield et al., 2009; Tapley et al., 2004). Studies using GRACE along with hydrological models and in situ observations have allowed for more accurate accounting of storages that are often difficult to measure such as groundwater (e.g., Famiglietti et al., 2011; Rodell et al., 2009) and drivers of ∆TWS such as evapotranspiration (Ramillien et al., 2006). Due to its coarse spatial resolution, although, GRACE is primarily applicable in regional‐ to global‐scale studies (e.g., Alley & Konikow, 2015), subregional scale ∆TWS studies have been accomplished through the integration of GRACE with hydrological models. For example, previous studies pursue this idea by calibrating GRACE data with hydrological models (Eicker et al., 2014; Werth & Güntner, 2010), assimilating GRACE data into hydrological models (Schumacher et al., 2016; Soltani et al., 2021), or downscaling GRACE data based on hydrological model predictions of ∆TWS with a higher spatial resolution (Vishwakarma et al., 2021; Zhong et al., 2021).

Alternatively, deformation data such as Global Navigation Satellite System (GNSS)‐derived vertical land motion measurements, assuming that they show the elastic solid Earth response to water mass loss and gain, can be used to generate ∆TWS maps with a spatial resolution on the order of tens of kilometers if the station density is high. Previous studies have employed Green's functions for a spherical, gravitating, Earth model (Farrell, 1972) such as the Preliminary Reference Earth Model (PREM; Dziewonski & Anderson, 1981), to either forward‐calculate the predicted deformation response to mass change (e.g., Carlson et al., 2020; Wahr et al., 2013) or to invert vertical displacements in order to solve for a change in water mass (e.g., Argus et al., 2017; Fu et al., 2015).

Studies have shown good agreement between GRACE and vertical GNSS observations using the PREM model, especially where deformation is dominated by large, regional TWS fluctuations (e.g., Chanard et al., 2014; Davis et al., 2004; Fu & Freymueller, 2012; Tregoning et al., 2009) and when GNSS processing errors are minimized (e.g., Chanard et al., 2020; Tregoning et al., 2009). Although location‐specific elastic structures of the Earth have been shown to improve the fit between GNSS and GRACE, this improvement is small in the vertical direction and so often the simpler PREM model is considered sufficient (e.g., Chanard et al., 2014). On the other hand, the choice of a more realistic three‐dimensional Earth model, as opposed to a simpler elastic half‐space model, has been shown to significantly impact the result (e.g., Argus et al., 2017; Chanard et al., 2014).

Several studies have solved for ∆TWS by inverting GNSS station displacements in California, benefitting from high station density and large annual amplitudes as well as steep multiyear trends due to recurrent drought (Argus et al., 2014, 2017; Borsa et al., 2014; Enzminger et al., 2019; Johnson et al., 2017). For example, Argus et al. (2017) used GNSS vertical displacements and estimated that the Sierra Nevada Mountains lost 45 km^3^ of water from October 2011 to October 2015. Borsa et al. (2014) also used vertical GNSS displacements across the Western United States and found a loss of more than 400 mm‐equivalent water height in March of 2014 near the Central Valley of California. Enzminger et al. (2019) used GNSS vertical displacements and focused on annual ∆TWS in the Sierra Nevada Mountains, finding that although multiyear variability exists, annual water storage change in the Sierra Nevada Mountains is ∼1 m‐equivalent water height. However, studies have also shown poor agreement between ∆TWS estimated from GRACE and GNSS in California (Argus et al., 2014, 2017). This may be due to differences in the spatial sensitivities of GRACE (regional/long‐wavelength) and GNSS (local/short wavelength), processing or inversion parameter choices, additional sources of error in the GNSS station time series, or leakage of GRACE from land into the ocean or between regions of high and low water mass change.

Another likely explanation is the overprinting of nonhydrologic loading signals on the GNSS time series in California, especially ongoing active tectonic processes. This is a challenge for accurately estimating ∆TWS in California due to the proximity of the Pacific/North American Plate boundary. Uplift of the Sierra Nevada Mountains (Hammond et al., 2016), San Bernardino mountains (Smith‐Konter et al., 2014), Coastal Ranges (Hammond et al., 2016), and Transverse Ranges (Johnson et al., 2020) is ongoing, as well as uplift in northern California associated with the Cascadia Subduction Zone (Yousefi et al., 2020), tectonic subsidence and uplift in Southern California (Johnson et al., 2020; Smith‐Konter et al., 2014), postseismic effects from large earthquakes including the 2010 M_W_ 7.2 El Mayor‐Cucapah earthquake in Southern California (Guns & Bennett, 2020; Rollins et al., 2015), and interseismic deformation, particularly along the San Andreas Fault (e.g., Smith‐Konter et al., 2014). All aforementioned deformation signals cannot be easily differentiated from hydrologic loading signals. Thus, GNSS alone may be insufficient to resolve long‐term ∆TWS in California and in other tectonically active regions because of the contamination from nonhydrologic loading signals.

**Figure 1:**
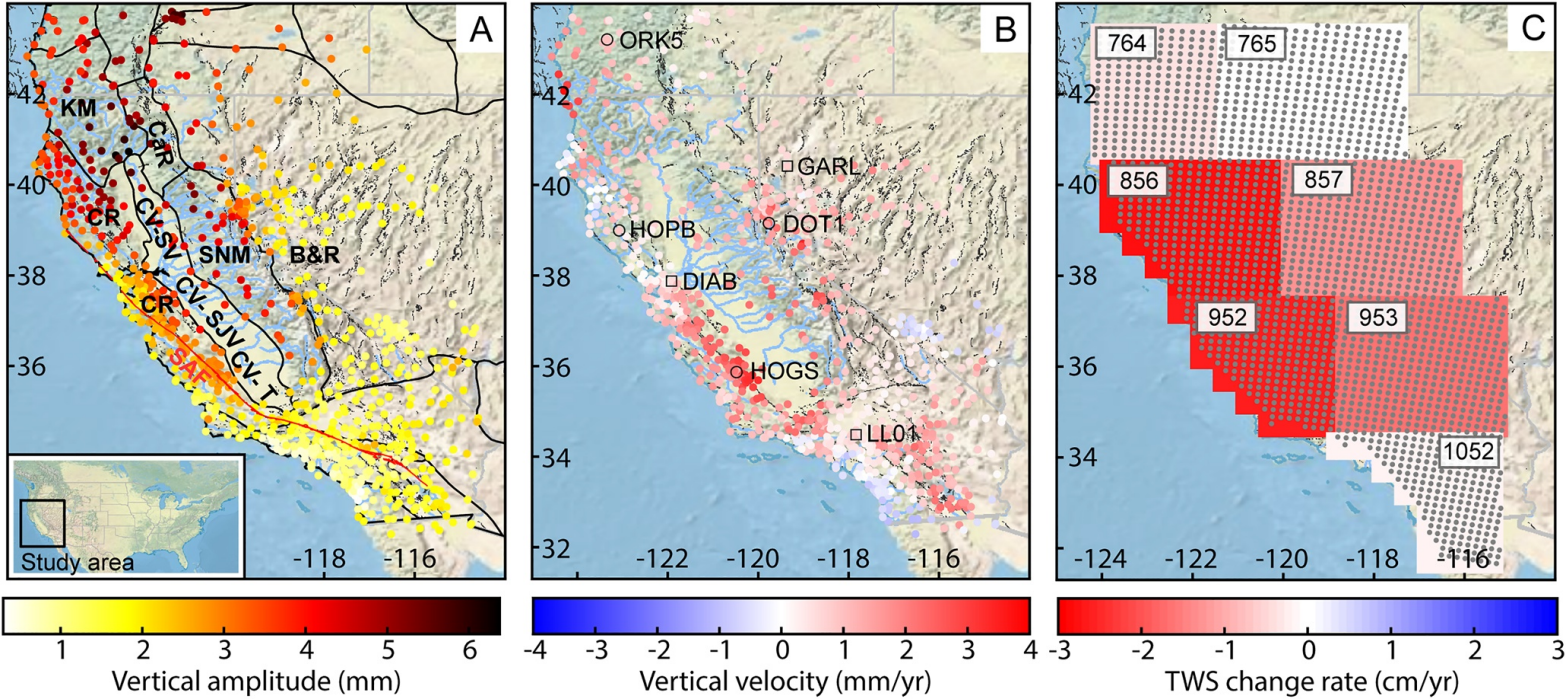
(a) Study region and annual amplitude of vertical deformation at Global Navigation Satellite System (GNSS) stations. GNSS stations colored to annual amplitude (mm) calculated from January 2003 to December 2016. Physiographic provinces labeled in bold (CSZ = Cascadia Subduction Zone; KM = Klamath Mountains; CaR = Southern Cascades Range; CV‐SV = Central Valley‐Sacramento Valley; CV‐SJV = Central Valley‐San Joaquin Valley; CV‐T = Central Valley‐Tulare Basin; B&R = Basin and Range; SNM = Sierra Nevada Mountains; and CR = Coastal Ranges) downloaded from the US Geological Survey (https://water.usgs.gov/lookup/getspatial?physio). Faults are in black and the San Andreas Fault (SAF) is outlined and labeled in red from the USGS faults database (https://www.usgs.gov/programs/earthquake-hazards/faults). Major rivers are in blue from the National Hydrography Data Set (https://data.cnra.ca.gov/dataset/national-hydrography-dataset-nhd/resource/510abd22-f63b-4981-a17e-3c76cec5fa18?inner_span=True). State outlines are in gray. Inset map shows the United States with the study region outlined in black. (b) GNSS stations colored to vertical velocity (mm/yr) calculated from January 2003 to December 2016. Station locations circled and labeled in black are used in Figure 2. Square station locations labeled in black are used in Figure 3. (c) Gravity Recovery and Climate Experiment Jet Propulsion Laboratory mascon cells colored to the rate of terrestrial water storage change in cm equivalent water height per year. Gray points show the inversion grid. A weighted median spatial filter is applied to GNSS station velocities in (b) and annual amplitudes in (a) using Delaunay triangulation similar to Hammond et al. (2016).

Poroelastic deformation signals are also overprinted on the hydrologic elastic loading signal. Deformation above aquifer systems is often much larger and of the opposite sign as the elastic response (Argus et al., 2017; Carlson et al., 2020). As groundwater is lost from an aquifer, pore space collapses, causing subsidence of the land surface. When aquifers are recharged, pore space opens, and uplift of the land surface occurs. The largest aquifer‐system in our study area is the Central Valley Aquifer, located between the Sierra Nevada Mountains to the east and the Coastal Ranges to the west (Figure 1a). In the Central Valley, and especially in the San Joaquin and Tulare Basins in the southern Central Valley, rapid subsidence has been observed due to groundwater withdrawals primarily to be used for irrigation of crops (Farr et al., 2015; Jeanne et al., 2019; Ojha et al., 2019). Other aquifer systems are found along the Pacific Coast and in the Basin and Range Province with significant historic and modern poroelastic deformation occurring, for example, in the Santa Ana Aquifer near Los Angeles, the Santa Clara Aquifer near San Francisco, and in the dry lake beds of the Mojave Desert (Blackwell et al., 2020; Chaussard et al., 2017; Riel et al., 2018; Solt & Sneed, 2014). Because the poroelastic response is opposite of the elastic response, GNSS stations showing poroelastic deformation cannot be included in elastic load inversions, resulting in a loss of information over areas of significant water loss, including the Central Valley Aquifer. Argus et al. (2017) attempted to remedy the data gap over the Central Valley using a priori estimates of groundwater loss from the Central Valley Hydrologic Model (Faunt et al., 2015) and Xiao et al. (2017). However, jointly incorporating direct observations of groundwater loss in hydrologic elastic loading inversion studies remains unaddressed in the literature. Other signals that may be superimposed on the GNSS time series are described in Chanard et al. (2020), including thermoelastic deformation and inadequate correction models applied in GNSS processing, such as those related to the GNSS draconitic period.

Merging GNSS and GRACE has the potential to generate more reliable ∆TWS estimates. Early attempts at a GRACE and GNSS combination were made to improve spatiotemporal coverage of TWS as well as spectral coverage, especially in the low‐degree harmonics, which were not well resolved with GRACE alone. These studies primarily focused on global‐scale TWS fluctuations and were performed by integrating the two geodetic data sets in the spherical harmonic domain. One of the earliest investigations of a joint inversion of GNSS and GRACE was performed by Kusche and Schrama (2005), who found that the inclusion of GNSS will have a more significant impact on the lower‐degree spherical harmonics. Wu et al. (2006), Rietbroek et al. (2009), and Rietbroek et al. (2012) included modeled ocean bottom pressure as well as GNSS and GRACE in a joint inversion and attempted to tackle the challenges posed by heterogeneous global GNSS station density using improved inversion techniques, a priori information from geophysical model data, and appropriate truncation of the spherical harmonic series. These early joint inversion studies worked well to resolve geocenter motion and large‐scale periodic mass loading signals, but higher spherical harmonics were found to contain large errors, especially in regions where GNSS station density was low. In some regions, an increase in the density of quality GNSS observations presents a new opportunity for a combination of GRACE and GNSS to be used to improve the spatial resolution of ∆TWS estimates beyond that of GRACE alone. Recent studies have constrained GNSS inversion results using GRACE‐derived ∆TWS by, for example, generating synthetic GNSS stations with GRACE‐derived displacements (Fok & Liu, 2019) or using GRACE as a large‐scale constraint in the inversion, minimizing the difference between inversion results and GRACE mass concentration solutions (Adusumilli et al., 2019). In the latter study, fit to either data set is not shown, which is an important validation of the inversion scheme. This is especially true for the case of GNSS inversion schemes that use Laplacian smoothing constraints because strong smoothing may produce a reasonable result but may have a poor fit to the data.

In this study, using a unified time‐dependent stochastic model, we jointly invert vertical displacement time series from GNSS stations across California and western Nevada and GRACE mascon products from NASA's Jet Propulsion Laboratory (JPL; Watkins et al., 2015) for ∆TWS. We focus on 14 years during the GRACE period of operation (January 2003–December 2016), which contains both drought and nondrought periods. We first invert only the GNSS vertical displacements to solve for monthly ∆TWS. Next, we combine GRACE and GNSS data in a joint inversion to solve for monthly ∆TWS using the JPL mascon data products. Finally, we compare the results from both inversions to GRACE mascon products from the Center for Space Research at the University of Texas at Austin (CSR; Save, 2020; Save et al., 2016), gridded TWS anomalies from the International Combination Service for Time‐variable Gravity Fields (COST‐G; Boergens et al., 2020), the WaterGap Global Hydrological Model (WGHM), and the Global Land Data Assimilation System, and the Catchment Land Surface Model (GLDAS‐2.1, CLSM). Both the GRACE mascon data and GNSS station locations are shown in Figure 1.

## 2 Data

### 2.1 GNSS Displacements

We obtain 1096 GNSS station time series from the Nevada Geodetic Laboratory's daily tenv3 solutions in the IGS14 reference frame (Blewitt et al., 2018). These solutions are produced using GipsyX software. Tropospheric delays are corrected using gridded VMF1 tropospheric products from the Vienna Mapping Function. The solid Earth tide, pole tide, ocean tidal loading, and ocean pole tide loading are also corrected according to IERS (2010) conventions. Nontidal loading corrections are not applied. Additional GNSS processing information can be found on the Nevada Geodetic Laboratory website (http://geodesy.unr.edu/gps/ngl.acn.txt). Although an inversion using both horizontal and vertical deformation may generate a more reliable inversion result, we choose to use only the vertical component of each time series because the horizontal component is dominated by tectonic deformation in California. We choose only stations that are within the grid boundary (Figure 1b) and have a minimum observation length of 2.5 years (according to Blewitt & Lavallée, 2002). Of these stations, 147 are dominated by the poroelastic response to groundwater loss, which is opposite of the elastic response to mass loss. We do not attempt to separate the poroelastic and elastic signals for each station and so take a conservative approach to station removal to ensure remaining stations primarily show the elastic response to mass change. Stations dominated by poroelastic deformation are identified based on location within the Central Valley Aquifer boundary and if the time series show a rate of subsidence faster than −2.5 mm/year similar to Hammond et al. (2018) or have annual amplitudes that are larger than 2 mm and peak from March 1 to May 31, when groundwater levels rise and we expect elastic displacement to be at a minimum, similar to Argus et al. (2017). In addition, 52 stations are dominated by volcanic deformation and so are removed. These stations are identified according to their location on or near caldera boundaries defined by the United States Geological Survey Volcano Hazards Program (https://www.usgs.gov/volcanoes/). In the San Francisco Bay area, 19 stations are dominated by rapid sediment compaction and tectonic subsidence and thus are also removed. Figure S7 in Supporting Information S1 shows the removed stations. After removing the stations for the reasons stated above, we are left with 878 time series. Because not all time series have consistent recording periods; for each time step in the inversion, there are a different number of GNSS stations used.

We correct all time series for glacial isostatic adjustment (GIA) using predictions of vertical velocity from ICE6G_D (VM5a; Peltier et al., 2018; Figure S8 in Supporting Information S1). In addition, we correct time series for the effects of nontidal atmospheric loading and nontidal ocean loading using vertical deformation estimates from the Earth‐System‐Modeling Group at the German Research Center for Geosciences (GFZ) given as 0.5° × 0.5° gridded solutions sampled every 3 hr (Dill & Dobslaw, 2013) averaged to daily to match the GNSS time series.

To correct the GNSS time series for steps, we first identify the steps using a step catalog provided by the Nevada Geodetic Laboratory. This catalog contains both coseismic offsets as well as antenna, receiver, or firmware changes that may cause steps in the time series. We identify additional steps that might be missing from the step catalog using a threshold of 4 cm or temporal gaps larger than 60 days between observations.

Once steps are identified, using a least squares approach, time series are fit with a long‐term trend, calculated using the weighted mean trend between steps, and annual and semiannual sinusoids. The linear rate and seasonal sinusoids are removed in order to isolate the noise, steps, and residuals from the long‐term and seasonal fit. We then use a continuous wavelet transform (CWT)‐ based approach to remove steps and reduce the noise of the time series. The equation for the forward CWT is given by (Torrence & Compo, 1998):

(1) $$\gamma \left(s,\tau \right)=\int f\left(t\right)\psi {*}_{s,\tau }\left(t\right)dt$$

where, * signifies the complex conjugation, *f(t)* is the time series, *t* is time, $\psi_{S,\tau }\left(t\right)$ are the wavelet basis functions for various scales, *s* (analogous to frequencies in a Fourier transform), and translations, τ, and γ are wavelet coefficients. The wavelet basis functions are generated from the mother wavelet, ψ(t), which, in our case is the Derivatives of Gaussian (DOG) mother wavelet, using (Torrence & Compo, 1998)

(2) $$\psi_{S,\tau }\left(t\right)=\frac{1}{\sqrt{s}}\psi \left(\frac{t-\tau }{s}\right)$$

Finally, the time series is reconstructed using the inverse CWT (Torrence & Compo, 1998):

(3) $$f\left(t\right)\;=\;\iint \gamma \left(s,t\right)\psi_{S,\tau }\left(t\right)d\tau ds$$

**Figure 2:**
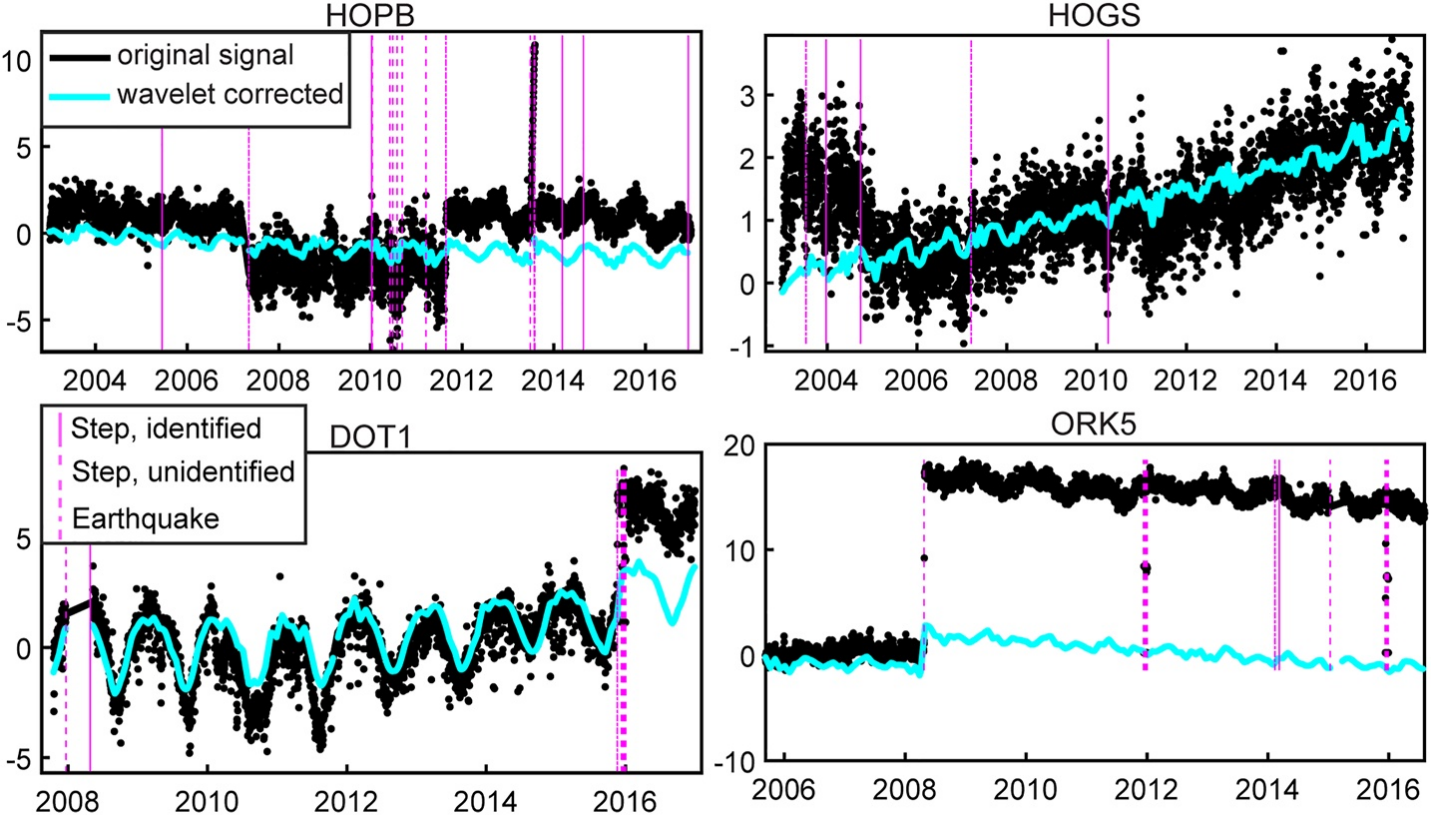
Examples of step‐corrected time series. Station locations are circled and labeled in black in Figure 1c. Black time series show the original Global Navigation Satellite System time series, and cyan shows the time series after the Continuous Wavelet Transform (CWT)‐based step correction and denoising. All time series are given in cm of vertical displacement. Vertical magenta lines show steps that were identified in the step catalog (solid line), steps not included in the step catalog that were identified because there is greater than 4 cm between observations or temporal gaps greater than 60 days (dashed line), and earthquakes identified in the step catalog (dash‐dot line). A constant initial value is removed from each time series such that the beginning of the time series is set to zero. In some rare cases when the step is very large, the step correction does not correct the complete step (ORK5); however, this minimally impacts the final result after the median spatial filter is applied.

To identify and remove steps, we exploit the rationale that Heaviside‐type functions cannot be reconstructed with a finite number of wavelet scales, as is the case for most transformations with smooth base functions. After removal of the trend and seasonal components, we perform the forward wavelet transform on the time series that contains mostly noise, subseasonal and interannual periods, and the step. The number of scales is identified so that the longest extracted period has the time series length (Torrence & Compo, 1998). We can apply the difference between the reconstructed and original time series to determine and subtract the steps from the time series. To further denoise the time series, we apply a soft thresholding scheme. Assuming that $\gamma_{s}$ is the continuous wavelet coefficient at the scale S, the denoised wavelet coefficient ${\hat{\gamma }}_{s}$ is obtained by (Gao et al., 2010)

(4) $${\hat{\gamma }}_{s}=\text{sgn}\left(\gamma_{s}\right)\max\left(0,\;\left|\gamma_{s}-\frac{M\sqrt{2\;\ln\left(N\right)}}{0.6745}\right|\right)$$

where |.| is the absolute value operator, sgn is the sign function, and M is the median absolute deviation of the wavelet coefficient at the scale S. After denoising the coefficients, we apply the inverse wavelet transform to reconstruct the denoised time series. We then add back the previously determined rate and seasonal components to the denoised and step‐corrected time series. Although the CWT‐based step correction does correct earthquake steps, it does not correct for postseismic or interseismic vertical deformation. In some cases, the wavelet‐based step correction also removes long‐period signals or does not fully correct large steps. This occurs at a similar rate as a conventional step correction method (see Text S3 and Figure S4 in Supporting Information S1) and has little impact on the final inversion results. Example step‐corrected and denoised time series are shown in Figure 2 and a comparison between our method and a conventional step correction method is shown in Figure S4 in Supporting Information S1.

One of the most significant transient vertical signals is postseismic deformation from two earthquakes greater than magnitude 7: the 2010 M_W_ 7.2 El Mayor‐Cucapah earthquake impacting 401 stations in Southern California and a 7.2 earthquake in 2005 occurring off of the coast of Northern California and impacting 70 stations. In addition to applying the wavelet‐based filter, time series of stations affected by postseismic deformation from these earthquakes are fit with a logarithmic function, $A\;\log\left(1+\frac{t}{\dot{\tau }}\right)$, where A is the amplitude term, *t* is time in years, and $\dot{\tau }$ is a time decay term which is allowed to vary between 0.77 ± 0.2 years (Klein et al., 2019). This is then removed from the affected time series. We find that much of the deformation from other earthquakes that occur during this time period are sufficiently removed during the denoising step. In a final step, we average GNSS displacements across each month to match the temporal sampling of the GRACE mascon solutions. For each time step in the inversion, we also apply a weighted median spatial filter to further smooth the signal in space, similar to Hammond et al. (2016).

### 2.2 GRACE Observations

We use GRACE mascon solutions from NASA's Jet Propulsion Laboratory (version RL06M) over the period of January 2003‐ December 2016 (Watkins et al., 2015; Wiese et al., 2016, 2018). The JPL mascon solutions are equal‐area 3° spherical cap mass concentration blocks that are estimated using intersatellite range rate measurements and given in units of cm‐equivalent water height. This mascon solution contains a priori conditioning to reduce striping based on altimetry observations and geophysical models (Watkins et al., 2015). The degree‐1 spherical harmonic coefficients are calculated according to Sun et al. (2016) and Swenson et al. (2008). In addition, the solutions are corrected for leakage errors that are common along coastlines using the Coastal Resolution Improvement filter, which helps ensure that the larger terrestrial signal is distributed only over the land area and the smaller ocean signal is maintained over the ocean (Watkins et al., 2015). The JPL mascon solutions are corrected for glacial isostatic adjustment using ICE6G‐D (Peltier et al., 2018), matching the GIA correction applied to the GNSS observations. Missing months in the GRACE observations are estimated using linear interpolation.

We also compare our joint inversion result with mascon solutions from the Center for Space Research at the University of Texas at Austin (Save, 2020; Save et al., 2016) and gridded TWS anomalies from the International Combination Service for Time‐variable Gravity Fields (Boergens et al., 2020). Unlike the JPL mascon solutions, the CSR mascon solutions do not use any external models as constraints. Mascons are related to the intersatellite range rate using a spherical harmonic expansion truncated at degree and order 120 (Save et al., 2016). The spatial sampling of the CSR solutions is approximately 1° of equatorial longitude, estimated on a hexagonal geodesic grid. The COST‐G solutions are an ensemble of GRACE spherical harmonic solutions from different processing centers including GFZ, Astronomical Institute at the University of Bern, Centre National d’Etudes Spatiales, Institute of Geodesy at the Graz University of Technology, and CSR. The COST‐G project aims to generate more reliable GRACE solutions with lower noise levels than individual solutions. Combinations are carried out on the normal equation level when available, otherwise, solutions are combined at the spherical harmonic coefficient level (Jäggi et al., 2020). This level‐3 product is derived from the low‐pass filtered ensemble spherical harmonic solution applying the VDK decorrelation approach (Boergens et al., 2020) and is given in terms of equivalent water height and provided on a 1° grid. We chose to use the JPL mascon solutions in the joint inversion as opposed to the other two solutions listed above because (a) the JPL mascon solutions have a resolution that matches the native GRACE resolution and (b) each observation contains an associated error estimate, which is necessary to perform the joint inversion and not currently available for the CSR mascon solutions. However, as will be shown in Sections 4 and 5, the three GRACE products show very similar ∆TWS and are highly correlated in space and time, as one would expect, and so the choice of mascon solution will have little impact on the final joint inversion result.

### 2.3 Hydrological Models

In addition, we compare our inversion results with two hydrological models: The WaterGap Hydrologic Model (WGHM) from the University of Frankfurt and the Global Land Data Assimilation System and Catchment Land Surface Model (GLDAS‐2.1, CLSM) from NASA Goddard. Both are global scale, climate‐driven hydrological models. We retrieve simulations from WGHM version 2.2d, which incorporates rainfall‐runoff routing, land use, water use, and water storage for all known components in a conceptual formulation of the water cycle. The model is forced with climate data and calibrated with river discharge observations to estimate water storage change as described in (Döll et al., 2014; Müller Schmied, 2017; Müller Schmied et al., 2016). It is provided at a spatial resolution of 0.5° and monthly temporal resolution. The GLDAS‐CLSM is a land‐surface model that simulates the Earth's water and energy cycle through meteorological forcing with subsequent assimilation of relevant satellite and terrestrial data within NASA's GLDAS framework (Beaudoing & Rodell, 2016; Rodell et al., 2004). The model includes a routine for shallow, unconfined aquifer layers but not for deeper or confined aquifer units. We retrieve water storage change output at monthly temporal resolution on a 1° grid.

## 3 Methods

### 3.1 GNSS‐Only Inversion Method

We assume a change in surface mass causes an elastic deformation effect that is recorded by GNSS vertical displacements (Farrell, 1972; Wahr et al., 2013). The relationship between vertical surface displacement at *n* GNSS stations, $L\;=\;{\left[L_{1},\;L_{2},\text{…},L_{n}\right]}^{T}$, and ∆TWS in equivalent water height calculated for *m* grid cells, $x\;={\left[x_{1},x_{2},\text{…},x_{m}\right]}^{T}$ is determined using a weighted iterative least squares approach. Each grid cell is approximated as a disk with a radius of 10 km (20 km grid spacing). We solve the following system of equations:

(5) L+v=Gx

Subject to the condition

(6) $${\left\|Pv\right\|}^{2}\to min$$

where, *P* is a diagonal weight matrix comprised of the inverse of the variance of the GNSS observations. G is an nxm matrix of Green's functions for a spherical, layered Earth based on disk radius, angular distance from the center of a load, and load‐Love numbers from the Preliminary Reference Earth Model (PREM; Dziewonski & Anderson, 1981) and $v={\left[v_{1},v_{2},\text{…},v_{n}\right]}^{T}$ are the observation residuals (Mikhail & Ackermann, 1982). To ensure the best‐fit solution varies smoothly between neighboring grid cells, we also consider the following constraint (Segall & Harris, 1986):

(7) λDx=0

where *D is* the Laplace operator and *λ* is a smoothing factor. Thus, Equation 7 is revised as the following:

(8) $$\;||Pv|{|}^{2}+\lambda^{2}||Dx|{|}^{2}\;\to \text{min}$$

where the second term defines the roughness of the solution. The optimal smoothing factor ($\hat{\lambda }$) provides a balance between model misfit and roughness and is found using trade‐off curves. The optimum solution ($\hat{x}$) is

(9) $$\hat{x}={\left(G^{T}PG+\hat{\lambda }D^{T}D\right)}^{-1}G^{T}PL$$

Equation 9 is applied to each time step to obtain a time series of water height for each grid cell. Next, we apply a linear Kalman filter to further reduce the temporal noise in the time series following (Shirzaei & Walter, 2010). Note that the term linear refers to the system dynamics, not the time series itself and the amount of smoothing is controlled by the variance‐covariance defined in Equation 11 (Grewal & Andrews, 2001). Consequently, we find that the RMS difference between the filtered and nonfiltered solutions are 0.020 m for the joint inversion and 0.029 m for the GNSS‐only inversion (Figure S3 in Supporting Information S1).

### 3.2 Uncertainty Estimation

The residual vertical displacements provide a measure of goodness of fit to the data. It is calculated at each GNSS station, using the equation

(10) v=Gx−L

Furthermore, to assess the uncertainty of the parameters, the standard errors can be calculated. The variance‐covariance matrix ($\Sigma_{x})\;$ for the optimal solution is (Mikhail & Ackermann, 1982)

(11) $$\Sigma_{x}\;=\;\sigma_{0}^{2}{\left(G^{T}PG+\hat{\lambda }D^{T}D\right)}^{-1}$$

where $\sigma_{0}^{2}$ is the variance factor given by

(12) $$\sigma_{0}^{2}=v^{T}Pv/df$$

where df is the number of degrees of freedom equal to the number of independent equations minus the number of unknowns. The diagonal elements of $\Sigma_{x}$ are the variance of the parameter and the square root of these elements provides us with a measure of the standard error of the ∆TWS at each grid cell.

### 3.3 GNSS and GRACE Joint Inversion Method

The formulation of a joint inversion framework is similar to the GNSS‐only inversion given by Equation 5. In this case, *L* =  ${\left[L_{D}\;L_{G}\right]}^{T}$ with $\;L_{G}$ comprising q GRACE mascon observations as $L_{G}=$ ${\left[L_{1},\;L_{2},\text{…},L_{q}\right]}^{T}$ and $L_{D}$ is a vector of n GNSS observations. The combined Green's function is rewritten as follows:

(13) $$G={\left[G_{D}\;A_{G}\right]}^{T}$$

where $G_{D}$ are the Green's functions from Section 3.1 and $A_{G}$ is a sparse matrix of size q×m as follows:

(14) $$A_{G}=\left[\begin{array}{lllll} \frac{1}{k_{1}} & \frac{1}{k_{1}} & \cdots & \frac{1}{k_{1}} & 0\\ 0 & 0 & \frac{1}{k_{2}} & \text{…} & \frac{1}{k_{2}}\\ \text{⋮} & \text{⋮} & \text{⋮} & \text{⋮} & \text{⋮}\\ 0 & \frac{1}{k_{q}} & \cdots & \frac{1}{k_{q}} & 0 \end{array}\right]$$

where, $k_{i}$, i=1:q is the number of grid cells within the i th GRACE mascon. The weight matrix is

(15) $$P=\left[\begin{array}{ll} \alpha \Sigma_{D}^{-1} & 0\\ 0 & (1-\alpha )\Sigma_{G}^{-1} \end{array}\right]$$

where $\Sigma_{D}$ and $\Sigma_{G}$ are the diagonal variance‐covariance matrices of the GNSS and GRACE observations, respectively, and α is a value between 0 and 1 that represents the relative weighting given to the GRACE and GNSS observations. Once again, we introduce roughness constraints (Equation 7) and minimize the sum of squared residuals and the model roughness according to Equation 8. The variance‐covariance matrix ($\Sigma_{x})\;$ is given in Equations 11 and 12.

### 3.4 Inversion Procedural and Parameter Choices

For both the GNSS‐only and the GNSS and GRACE joint inversions, we use a CWT to decompose the GNSS and GRACE time series into long‐term and short‐term components and invert both separately. We use Equations (1), (2), (3) to reconstruct the short‐term component of the time series by retaining only wavelet scales with periods less than 2 years. We then subtract the reconstructed short‐term component from the full reconstructed time series to obtain the long‐term component. The short‐term and long‐term parts of the signal are inverted separately and, given the linearity of the wavelet transform, the results are superimposed. In this way, we preserve the entire signal and maintain interannual variability in the short‐term and long‐term components. The wavelet‐based signal separation is shown in Figure 2a for GRACE mascon time series and in Figure 2b for GNSS station displacements. ∆TWS maps are generated for each month from January 2003 to December 2016.

In addition, we add boundary constraints for ∆TWS during drought periods for grid points located within the Central Valley Aquifer boundary to address the spatial observation gap for GNSS observations in this area. To compensate for the missing GNSS observations, we use groundwater storage trends calculated from January 2007‐ January 2010 based on estimates from Ojha et al. (2018) to set the upper and lower bounds in the inversion. The authors used vertical land motion from Interferometric Synthetic Aperture Radar (InSAR) in a 1‐D poroelastic model to estimate groundwater storage change in the Central Valley. We also assume that the distribution of groundwater storage change is similar during the 2012–2015 drought period and use groundwater storage change rate estimates from Ojha et al. (2019), which are found using the same poroelastic model with vertical land motion trends from InSAR and GNSS calculated throughout October 2011‐September 2015. Both studies were found to agree well with groundwater loss estimated by GRACE and other hydrological data sets. Additional discussion on the bound‐setting procedure can be found in the Supporting Information (Text S1 in Supporting Information S1).

**Figure 3:**
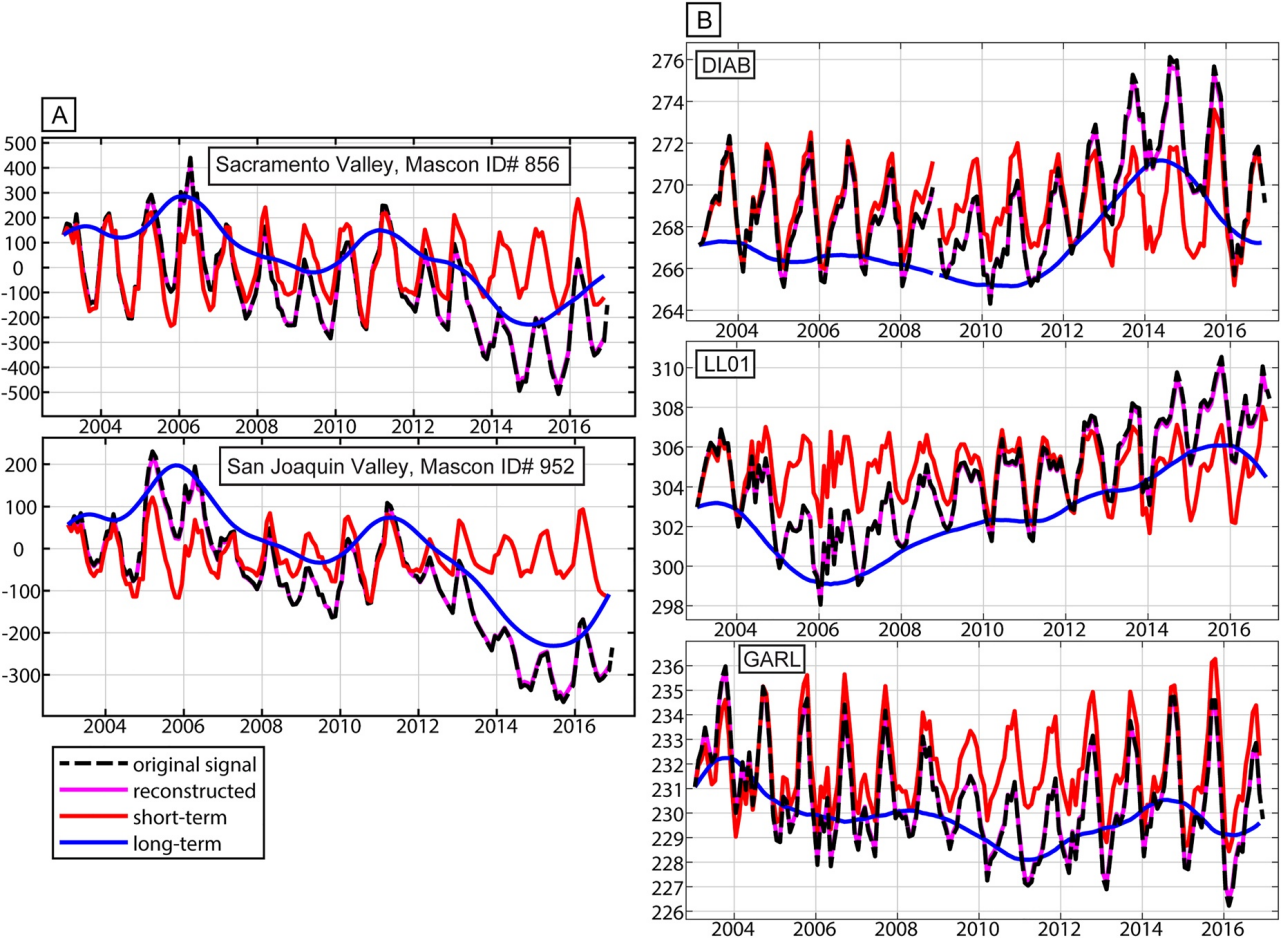
Examples of separated short‐ and long‐term signal components based on the Continuous Wavelet Transform (CWT) decomposition. For all panels black is the original time series, red shows the CWT‐determined short‐term component, blue shows the CWT‐determined long‐term component, and magenta shows the reconstructed time series (red + blue time series). (a) Gravity Recovery and Climate Experiment Jet Propulsion Laboratory mascon time series. The top panel is the mascon cell that covers the Sacramento Valley, and the bottom panel is the mascon cell that covers the San Joaquin Valley. Both time series are given in mm‐equivalent water height. (b) Global Navigation Satellite System (GNSS) station vertical displacement time series. The locations of the GNSS stations are indicated by a square and labeled in black in Figure 1c. All GNSS time series are given in mm. The GNSS short‐term time series are vertically offset so that they can be compared.

**Figure 4:**
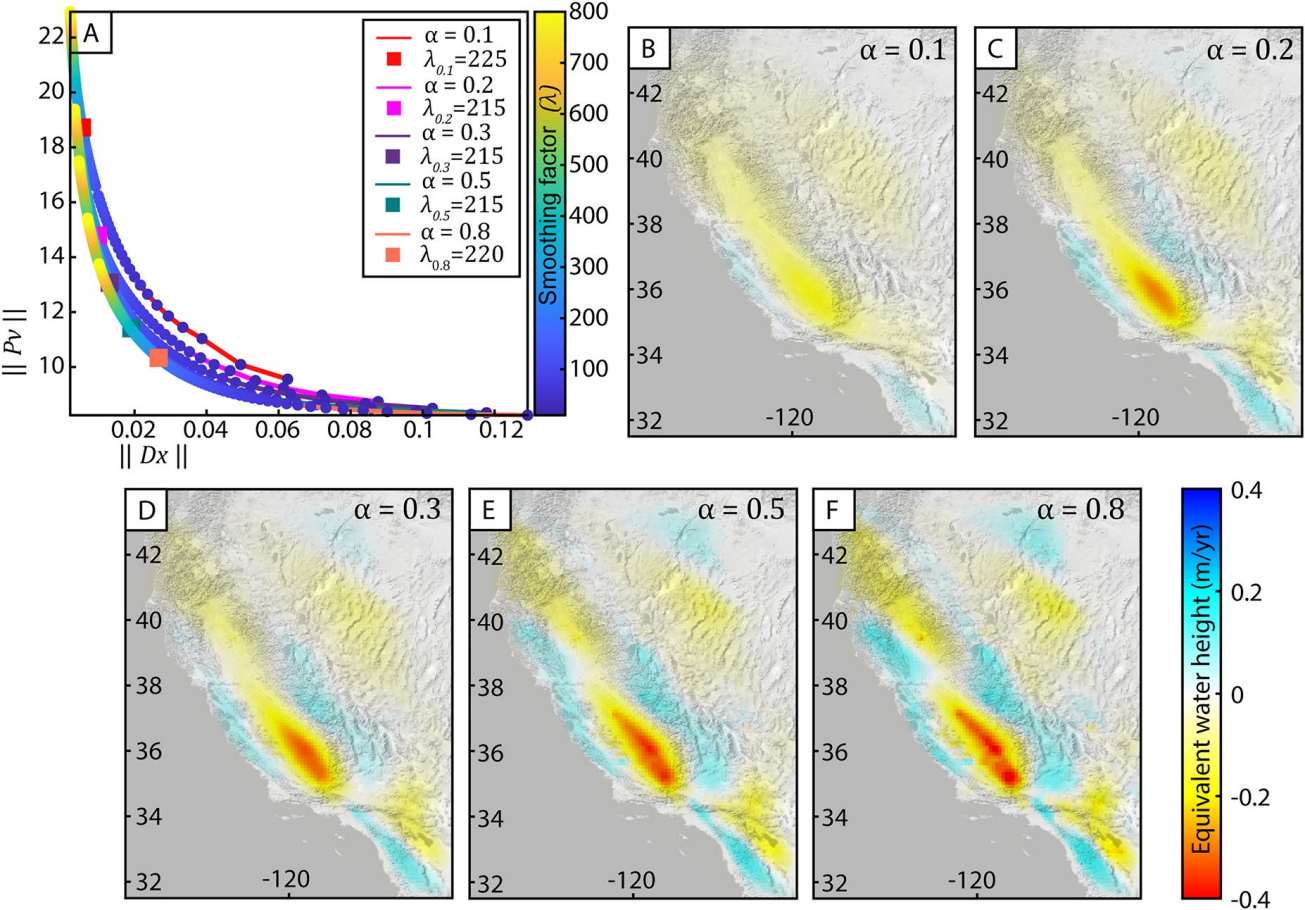
Example of inversion parameter selection over the epoch covering the first drought period (January 2007–January 2010) for the Global Navigation Satellite System and Gravity Recovery and Climate Experiment joint inversion. (a) Trade‐off curves for different values of α. Colored lines correspond to *α* = 0.1 (red), *α* = 0.2 (magenta), *α* = 0.2 (purple), *α* = 0.5 (dark green), and *α* = 0.8 (orange). Colored squares indicate the optimal λ value ($\hat{\lambda }$) for each α curve. (b)**‐**(f) Joint inversion results for TWS rate during the first drought period when (b) *α* = 0.1 and $\hat{\lambda }=225$ (c) *α* = 0.2 and $\hat{\lambda }=215$ (d) *α* = 0.3 and $\hat{\lambda }=215$ (e) *α* = 0.5 and $\hat{\lambda }=215$ (f) *α* = 0.8 and $\hat{\lambda }=220$.

Finally, we determine both the value of the relative weighting parameter, α, as well as the optimal smoothing parameter, $\hat{\lambda }$, using trade‐off curves. Higher values of λ produce an overly smooth, ill‐fitting result, while lower values of λ produce a patchy result that contains sharp, unrealistic transitions in TWS between grid cells. Lower values of α correspond to a higher relative weight given to the GRACE observations, while higher values of α correspond to a higher relative weight given to the GNSS observations. Generally, because the GRACE solutions are smooth, lower values of α correspond to a smoother model with a larger misfit with the GNSS observations, while higher values of α result in a rougher model. In order to select both values for the joint inversion, we run inversions for a range of λ values from 10 to 800 with a step size of 5 and for values of *α* ranging from 0.1 to 0.9 with a step size of 0.1. All α values greater than 0.6 produce results that are indistinguishable from one another. After inspecting the suite of inversion results and residuals and considering the fact that the long‐term GNSS observations may be contaminated by tectonic deformation, we choose a value of *α* = 0.3 for the long‐term component and *α* = 0.5 for the short‐term component. Examples of inversion results with different values of α are shown in Figure 4 for the rate of ∆TWS over the first drought period (January 2007–January 2010). It is clear that when GRACE is weighted higher, for example, when *α* = 0.1, the magnitude of water storage change is smeared across the study region. However, the solutions are still somewhat similar for all values of α with a mean RMS between solutions where *α* = 0.1 and where *α* = 0.8 of 0.037 m‐equivalent water height.

### 3.5 Synthetic Test

**Figure 5:**
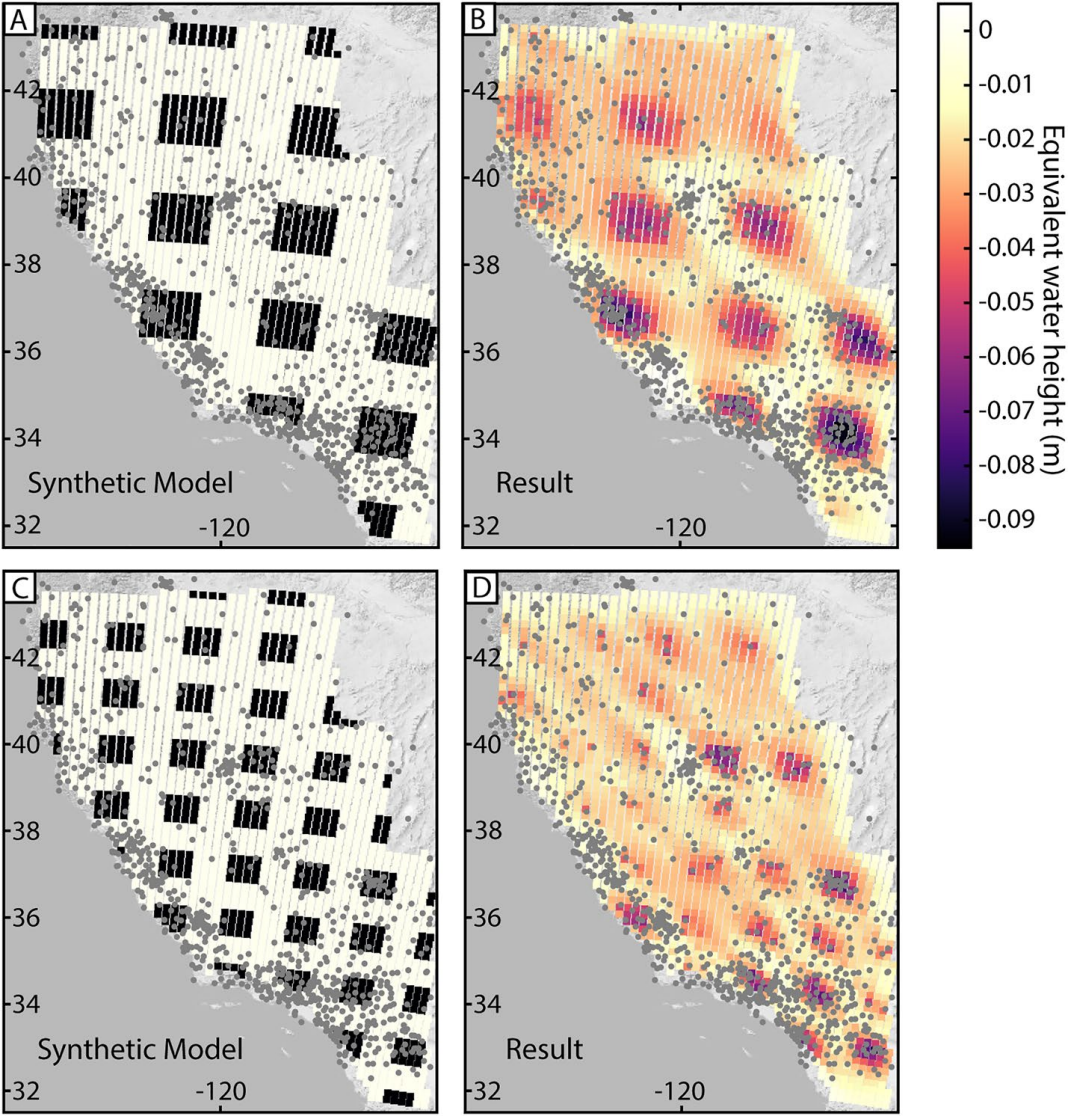
Synthetic tests. (a) Checkerboard synthetic model 140 × 140 km resolution and (b) results using the joint Global Navigation Satellite System and Gravity Recovery and Climate Experiment inversion method. (c) Checkerboard synthetic model 80 × 80 km resolution and (d) results.

In order to test the ability of our inversion method to resolve water mass change, we use two different synthetic mass distributions in a checkerboard pattern. For the first synthetic test, each mass has dimensions of 140 × 140 km and an equivalent water height of −0.1 m (Figures 5a and 5c). For the second synthetic test, each mass has dimensions of 80 × 80 km and an equivalent water height of −0.1 m (Figures 5b and 5d). Outside of the masses, the equivalent water height is 0. Uplift due to the input synthetic mass is forward calculated at the locations of GNSS stations. Synthetic GRACE data are the average of the mass within each mascon area. We set $\hat{\lambda }$ = 215 and *α* = 0.5. Using the joint inversion framework, we are able to resolve the masses well, especially near the center of the mass and where the GNSS station density is higher. The higher spatial resolution test shows that masses of this size can be resolved if the station density is high; for example, in southern California. There is some smearing between the masses and the edges of the masses, a consequence of applying the Laplacian operator in such optimization problems. Mean residuals between the input map of ∆TWS and output ∆TWS is 0.03 and 0.022 m for the 80 × 80 and 140 × 140 km resolution synthetic tests, respectively.

## 4 Results

### 4.1 ∆TWS Results and Agreement With GRACE Solutions

**Figure 6:**
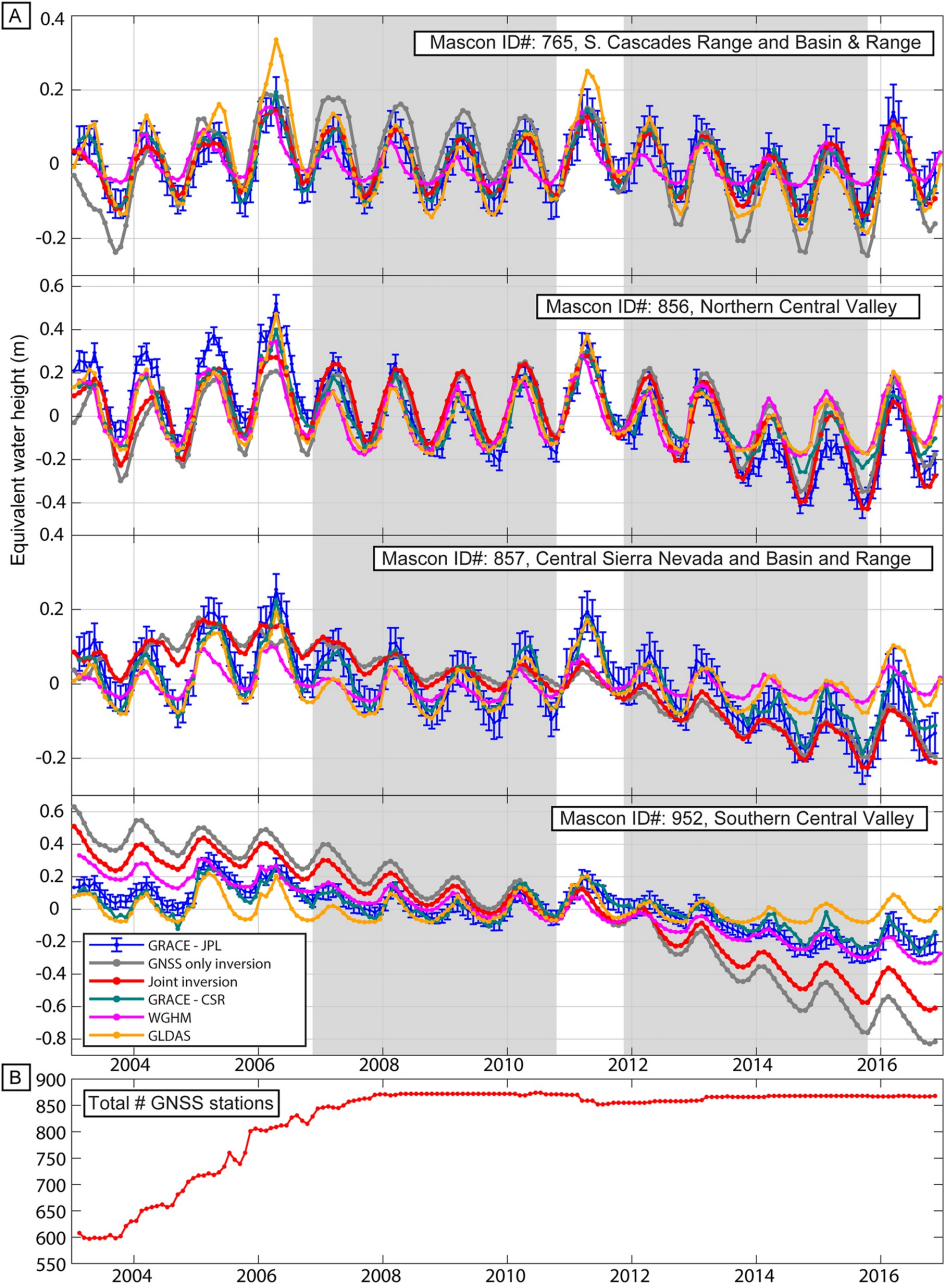
(a) Time series of terrestrial water storage change averaged over Jet Propulsion Laboratory (JPL) mascon regions. Blue time series are Gravity Recovery and Climate Experiment (GRACE) JPL mascon solutions with vertical bars showing the standard error, red time series are Global Navigation Satellite System (GNSS) and GRACE joint inversion results, gray are the GNSS‐only inversion results, green are the GRACE CSR mascon solutions, purple are results from the WGHM hydrological model, and yellow are the GLDAS hydrological model. The top panel is the average over the JPL mascon covering the northern mountain ranges, the second is the average over the JPL mascon covering the Sacramento Valley, the third is the average over the JPL mascon covering the Basin and Range and Sierra Nevada Mountains, and the bottom panel shows the average over the JPL mascon covering the San Joaquin Valley and Tulare Basin. Gray shading shows drought periods. All time series are given in m‐equivalent water height and centered at the mean value. (b) Total number of GNSS stations included in each time step of the GNSS and GRACE joint and GNSS‐only inversions.

As has been found in previous studies, we find that the magnitude of water storage change using the GNSS‐only inversion framework is in many cases larger than GRACE ∆TWS alone (e.g., Argus et al., 2017) with the largest deviations occurring over the southern Central Valley Aquifer. Here, although each GRACE solution and both inversion results record two drought periods (water years of ∼October 2006–October 2009 and ∼October 2011–October 2015) and some drought recovery (wet seasons of 2010, 2011, and 2016), the magnitude of water storage loss and gain is not the same amongst different GRACE solutions and inversion results (Figure 6a). Elsewhere, when averaged over the GRACE JPL mascon cell areas, the inversion results and GRACE solutions agree well. This is particularly true over the northern Central Valley and northern mountain ranges (northern Coastal Ranges, Southern Cascades Range, and Klamath Mountains). Total water storage change over the two drought periods averaged over the mascon areas is given in Table 1. Over the second drought period, we also compare our results with those from Argus et al. (2017). This study uses GNSS stations to estimate water storage change. As expected, the Argus et al. (2017) solutions are similar to the GNSS‐only inversion results.

**Table 1:** Total Water Storage Change in Meter‐Equivalent Water Height During Drought Averaged Over JPL Mascon Areas

Drought 1	Mascon ID# 764	Mascon ID# 765	Mascon ID# 856	Mascon ID# 857	Mascon ID# 952	Mascon ID# 953	Mascon ID# 1052
GPS Only	−0.219	−0.045	0.103	−0.08	−0.303	−0.021	−0.087
Joint	−0.108	−0.033	0.017	−0.095	−0.24	−0.057	−0.055
GRACE‐JPL	−0.025	−0.039	−0.143	−0.111	−0.129	−0.098	0.012
GRACE‐COSTG	−0.03	−0.027	−0.069	−0.067	−0.082	−0.062	−0.024
GRACE‐CSR	−0.004	−0.013	−0.067	−0.055	−0.088	−0.076	−0.029
WGHM	−0.094	−0.026	−0.052	−0.02	−0.161	−0.005	−0.039
GLDAS	−0.016	−0.064	−0.006	−0.018	0.002	−0.001	0.006
Drought 2							
GPS Only	−0.489	−0.16	−0.265	−0.165	−0.656	0.009	−0.157
Joint	−0.258	−0.093	−0.326	−0.188	−0.493	−0.12	−0.087
GRACE‐JPL	−0.121	−0.117	−0.389	−0.229	−0.31	−0.221	0.009
GRACE‐COSTG	−0.114	−0.14	−0.177	−0.187	−0.205	−0.169	−0.077
GRACE‐CSR	−0.067	−0.111	−0.197	−0.159	−0.262	−0.165	−0.041
WGHM	−0.099	−0.031	−0.109	−0.035	−0.211	−0.019	−0.077
GLDAS	−0.104	−0.146	−0.087	−0.05	−0.028	−0.019	−0.006
Argus et al. (2017)	−0.374	−0.128	−0.361	−0.216	−0.686	−0.183	−0.203

*Note.* Water storage change calculated over the period of October 2006–October 2009 for the first drought period and October 2011–October 2015 for the second drought period.

**Figure 7:**
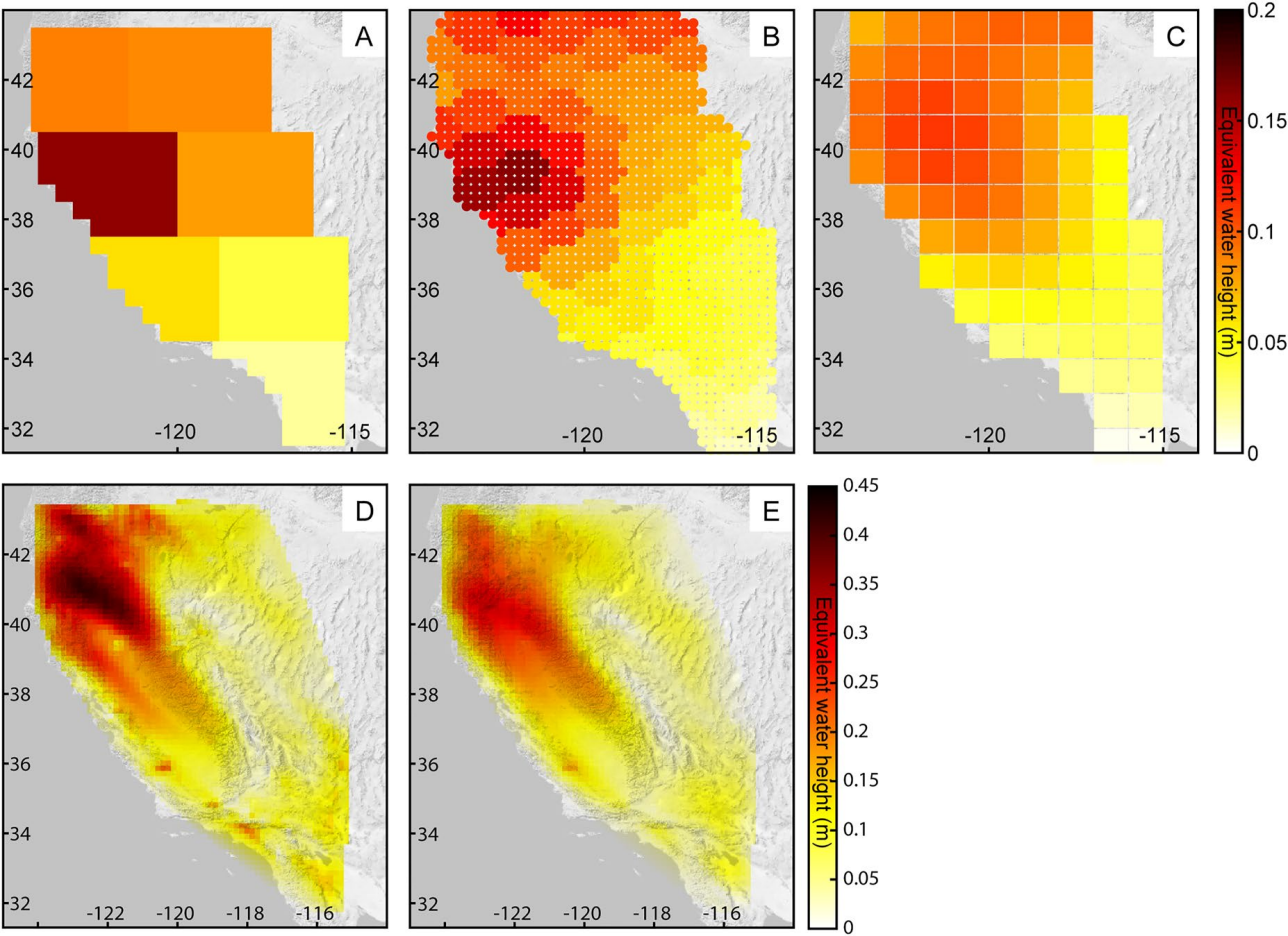
Annual amplitude of water storage change. (a) Gravity Recovery and Climate Experiment Jet Propulsion Laboratory (GRACE JPL) mascon solutions. (b) GRACE Center for Space Research mascon solutions. (c) GRACE COST‐G solutions. (d) Global Navigation Satellite System (GNSS)‐only inversion results. (e) GNSS and GRACE joint inversion results. The color bar to the right of (c) applies to subplots a, b, and c. The color bar to the right of (e) applies to subplots d and e.

In addition, the inversion results show larger annual water storage change than the GRACE solutions, found by fitting a sinusoid with a period of 1 year to each time series. The largest annual amplitude of water storage change occurs in the northern mountain ranges and northern Central Valley (Figure 7). The GNSS‐only inversion results show an annual amplitude of ∼0.4–0.45 m‐equivalent water height in the northern mountain ranges while the joint inversion results show an annual amplitude of ∼0.3 m‐equivalent water height in the northern mountain ranges. The GRACE JPL and GRACE CSR solutions show an annual amplitude of ∼0.2 m‐equivalent water height over the northern Central Valley and the GRACE COST‐G solutions show ∼0.1 m‐equivalent water height. The differences in annual amplitude found between the GNSS inversion results and GRACE solutions are consistent with previous studies for this region (e.g., Argus et al., 2014; Enzminger et al., 2019) and may be due, in part, to the large spatial resolution of the GRACE satellites. The satellites are unable to differentiate between regions of high and low mass change if the width of the region is less than ∼400 km, which is the case for the mountain ranges and Central Valley Aquifer in California. In addition, leakage between mascon areas may also be present. This effect may be the reason for the larger overall annual amplitude observed by GRACE over the mascon covering the Basin and Range (Mascon ID# 857) than by the inversion results. This mascon is located adjacent to the Sierra Nevada Mountains and northern Central Valley, which are both provinces with large annual amplitudes. Thus, signal from these regions with large annual ∆TWS may be leaking into the Basin and Range, which shows a low annual ∆TWS signal according to the inversion results.

Although the annual amplitude values in Figure 7 are found by fitting a single sinusoid with period of 1 year, the annual amplitude is in reality not constant from year to year. For example, we find that the annual amplitude of TWS was reduced by ∼10%‐30% across the Sierra Nevada Mountains during the second drought period as compared with the entire observation period according to both inversion results. The GRACE solutions show similar year‐to‐year variations in annual amplitude. The CSR mascon solutions show a reduction in the annual amplitude of TWS of ∼30%‐45% over the central Sierra Nevada Mountains, Central Valley, and south‐central Coast Ranges during the second drought period. The GRACE COST‐G solutions show a reduction of ∼30% in the San Joaquin Valley and southern Sierra Nevada Mountains and ∼20% in the Sacramento Valley and northern Sierra Nevada Mountains.

### 4.2 Spatiotemporal Variability of TWS Averaged Over the Physiographic Provinces

The physiographic provinces (Figure 1a) represent distinct geographic regions with different climates. The GRACE JPL mascon areas generally span two or more of these physiographic provinces and a single province may be covered by more than one mascon. Therefore, the GRACE JPL mascon products cannot be used to distinguish ∆TWS between physiographic provinces. The CSR mascon and COST‐G GRACE solutions are provided at a higher resolution and thus can be used to differentiate ∆TWS between physiographic provinces. However, the spatial sensitivity of the GRACE solutions is still considered to be ∼300–400 km.

A summary of ∆TWS calculated over the two drought periods averaged over the physiographic provinces can be found in Table 2. Although there is fairly good agreement between ∆TWS averaged over the area covered by the GRACE JPL mascons (with the exception of the mascon covering the southern Central Valley), differences between the GRACE solutions (CSR and COST‐G), hydrological models (WGHM and GLDAS‐CLSM), and inversion results are more significant over the physiographic provinces. The largest deviations between the inversion results and the GRACE solutions and hydrological models occur during the two drought periods in the Central Valley and Klamath Mountains.

**Table 2:** Total Water Storage Change in Meter‐Equivalent Water Height During Drought Averaged Over Physiographic Provinces

Drought 1	Sierra Nevada Mountains	Central Valley	Basin & Range	Southern Cascades Mountains	Klamath Mountains	Coastal Ranges
GPS Only	−0.029	−0.205	−0.026	−0.107	−0.326	−0.046
Joint	−0.018	−0.228	−0.049	−0.095	−0.206	−0.081
GRACE‐COSTG	−0.083	−0.084	−0.054	−0.051	−0.036	−0.07
GRACE‐CSR	−0.093	−0.093	−0.042	−0.02	−0.008	−0.061
WGHM	−0.037	−0.196	−0.013	−0.08	−0.095	−0.065
GLDAS	−0.017	−0.002	−0.028	−0.003	−0.023	0.008
Drought 2						
GPS Only	−0.336	−0.606	−0.09	−0.482	−0.622	−0.394
Joint	−0.329	−0.478	−0.114	−0.25	−0.352	−0.413
GRACE‐COSTG	−0.213	−0.208	−0.167	−0.151	−0.114	−0.175
GRACE‐CSR	−0.244	−0.27	−0.135	−0.12	−0.069	−0.192
WGHM	−0.116	−0.23	−0.052	−0.113	−0.106	−0.107
GLDAS	−0.065	−0.054	−0.072	−0.099	−0.088	−0.046
Argus et al. (2017)	−0.659	−0.692	−0.136	−0.308	−0.472	−0.378

*Note.* Water storage change calculated over the period of October 2006–October 2009 for the first drought period and October 2011–October 2015 for the second drought period.

**Figure 8:**
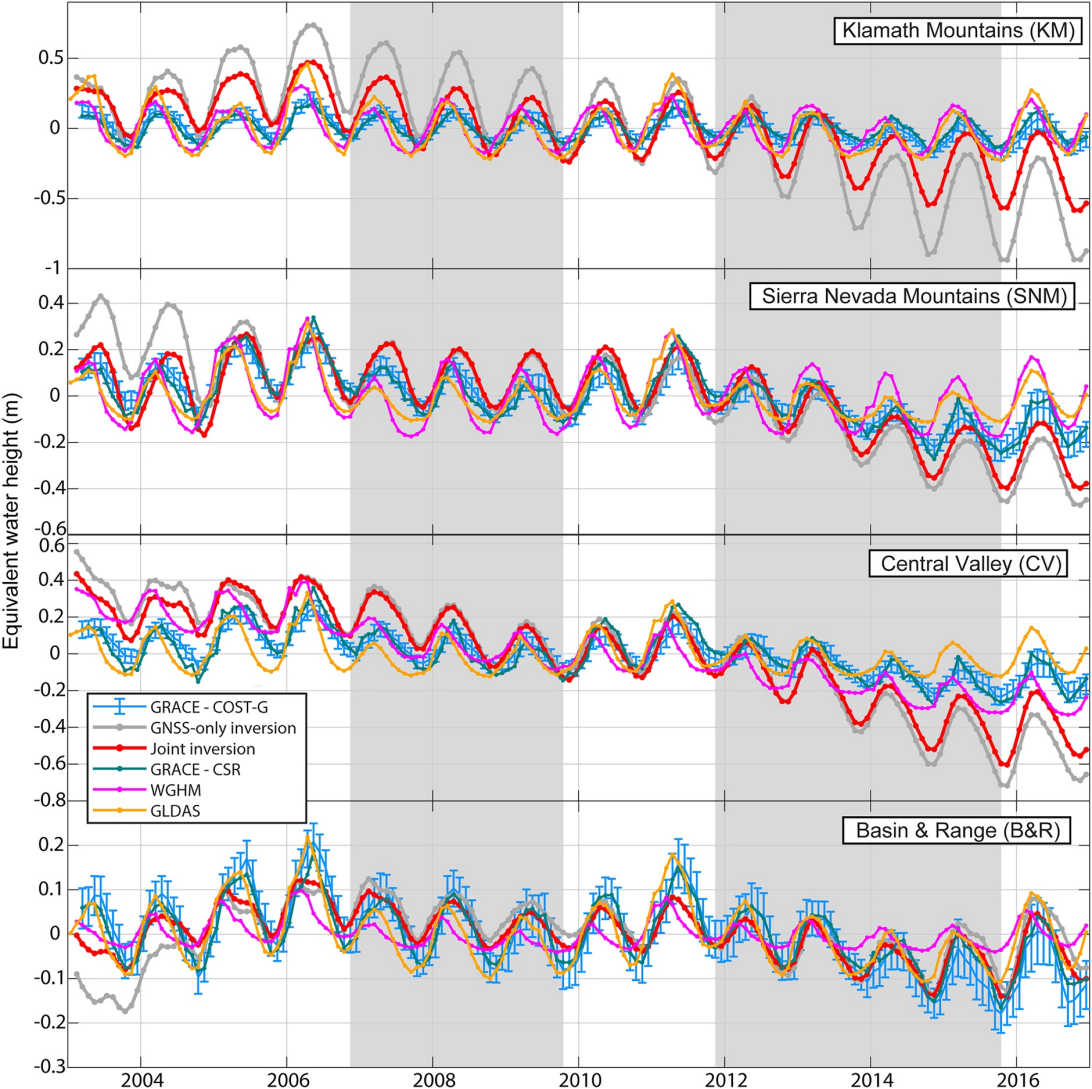
Time series of terrestrial water storage change averaged over physiographic provinces in Figure 1a. Blue time series are GRACE COST‐G mascon solutions with vertical bars showing the standard error, red time series are GNSS and GRACE joint inversion results, gray are the GNSS‐only inversion results, green are the GRACE Center for Space Research mascon solutions, purple are results from the WGHM hydrological model, and yellow are the GLDAS hydrological model. Gray shading shows drought periods. All time series are given in m‐equivalent water height and centered on the mean value of each time series.

The hydrological models only contain a conceptual formulation of groundwater (WGHM) or the shallow groundwater component (GLDAS). Thus, these models may not capture the total ∆TWS, particularly in the Central Valley where we know that anthropogenic groundwater removal from deeper aquifer layers is a significant driver of the ∆TWS rate (e.g., Ojha et al., 2018). Over the Sierra Nevada Mountains during the second drought period, the difference is nearly an order of magnitude between the largest average ∆TWS (GNSS‐only inversion results, −0.336 m‐equivalent water height) and smallest average ∆TWS (the GLDAS model, −0.065 m‐equivalent water height). Comparing Figure 6, which shows ∆TWS averaged over the mascon areas, and Figure 8, which shows ∆TWS averaged over the physiographic provinces, we can see that the inversion results agree better with the GRACE solutions over the mascon areas compared to over the physiographic provinces. This indicates that the inversion method better isolates mass change over the mountain ranges and Central Valley, minimizing leakage between physiographic provinces in California that is present in the GRACE solutions.

## 5 Discussion

### 5.1 GNSS and GRACE Joint Inversion Data Fit and Uncertainty Analysis

**Figure 9:**
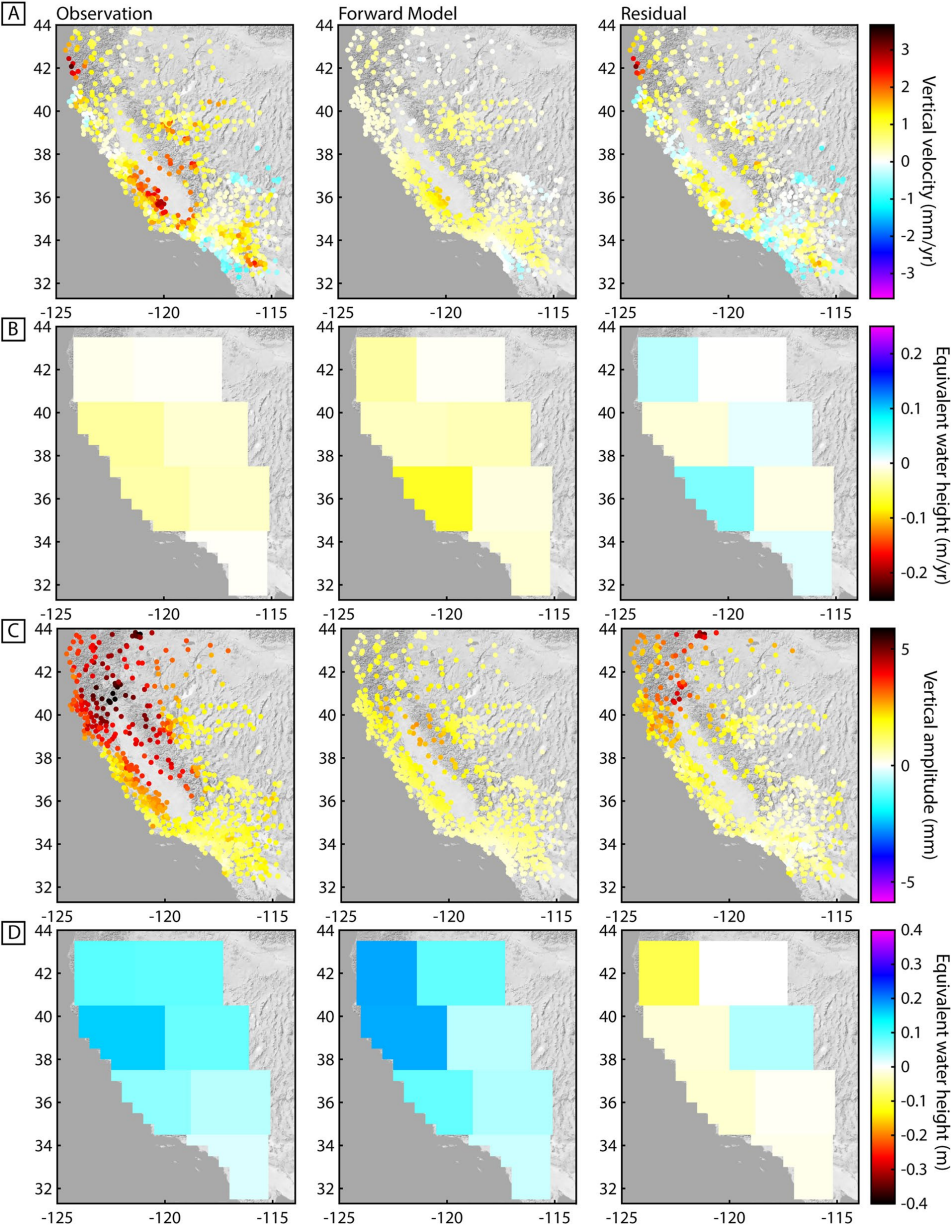
Global Navigation Satellite System (GNSS) and Gravity Recovery and Climate Experiment (GRACE) joint inversion residuals. (a) GNSS velocity residuals in mm/yr. (b) terrestrial water storage change (∆TWS) rate residuals in m‐equivalent water height/year. (c) Annual amplitude of GNSS residuals in mm. (d) ∆TWS annual amplitude residuals in m‐equivalent water height. First columns in (a–d) show the observations, second columns show the forward models, and the third columns are the residuals. Maximum and minimum uplift in (a), (c) approximately corresponds to water storage change in (b), (d) over a disc approximately the size of a mascon.

The GNSS and GRACE joint inversion residual vertical velocity and residual ∆TWS rate is shown in Figure 9. The joint inversion finds a balance between the GNSS data, which forces a larger ∆TWS, and the smaller GRACE ∆TWS. Thus, the vertical velocity residuals are dominantly positive, except in Southern California, and the residual ∆TWS rate calculated using the GRACE observations is also dominantly positive. The residual vertical deformation may primarily contain residual tectonic deformation in Southern California, along the San Andreas Fault, and near the Northern California coast, where GNSS vertical deformation is influenced by the Cascadia Subduction Zone. The mean standard error is 0.66 m‐equivalent water height and is dominantly below 0.7 m‐equivalent water height across space and time (Figure S10 in Supporting Information S1). The joint inversion overall has a lower error that the GNSS‐only inversion by ∼0.3 m‐equivalent water height. We also calculate the correlation coefficient and RMS between all solutions shown in Table 3. Unsurprisingly, the GRACE solutions are highly correlated and are also correlated with the GLDAS hydrological model. The joint and GNSS‐only inversion results are also highly correlated with low RMS. The largest RMS difference is between all solutions and the GNSS‐only inversion results.

In addition to formal uncertainty in the inversion, there may be some additional sources of error that may cause uncertainty in the results. For example, although we carefully remove stations dominated by sources of deformation not related to hydrologic loading, the overprinting of other deformation signals on our GNSS time series may bias the final inversion results. Minor sources of uncertainty may be present directly adjacent to aquifers and near the edges of our model grid where there may be some small influence from loads outside of our study region. Larger biases may occur due to tectonic deformation along the San Andreas Fault, near the Cascadia Subduction Zone, and especially in Southern California where the hydrologic loading signal is small and vertical tectonic deformation is significant. Because GRACE is not sensitive to present tectonic deformation, the long‐term component of the signal in the joint inversion results is less impacted by these sources of error than the GNSS‐only inversion; however, this does not mean the error is eliminated completely. Depending on the study region, some uncertainty may be reduced by using both horizontal and vertical deformation to invert for hydrologic loading if tectonic deformation is able to be effectively removed or if horizontal tectonic deformation is small. Although including horizontal deformation is outside of the scope of this work, it could be a topic of future work.

**Table 3:** RMS Difference and the Correlation Coefficient Between Solutions

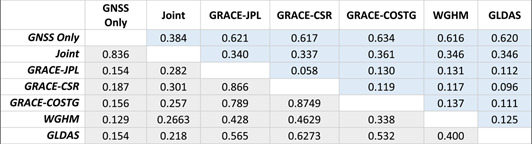

*Note.* Blue cells show average RMS between solutions in meter‐equivalent water height. Gray cells show the correlation coefficient between solutions.

Finally, we do not remove the effects of the draconitic error, which is suggested to possibly be a large source of error in GNSS station time series (Chanard et al., 2020) and could impact the final solution. The GNSS draconitic year has a period of ∼351.4 days, which is close to the annual period we expect from hydrologic loading signals (e.g., Chanard et al., 2020; Ray et al., 2008, 2013). Because there is so much year‐to‐year variability in annual and semiannual hydrologic signals, we cannot be confident in any separation of the two signals. Fu et al. (2015) looked at GNSS stations in Washington and Oregon, USA and found that the ratio between the draconitic component and the annual amplitude is ∼21% and the ratio between the second draconitic and semiannual amplitude is ∼22%. Although these ratios may not be exact for the reason stated above, these results indicate that the draconitic signal could be a significant source of error in seasonal hydrologic loading signals. However, since this study, reprocessing campaigns by both the Nevada Geodetic Laboratory (solutions used in this study) and JPL (as used in the Fu et al. study) have reduced the overall scatter in GNSS time series, likely indicative of improved precision over earlier GNSS processing campaigns (Martens et al., 2020), which may influence the estimated ratio between the draconitic and hydrologic components.

### 5.2 Sources of Agreement and Disagreement

Overall, we find regional agreement between the inversion results, GRACE CSR mascon and COST‐G solutions, and the WGHM and GLDAS hydrological models. In addition, the joint inversion results successfully balance the lower magnitude, smoother ∆TWS observations from the GRACE solutions with improved resolution of GNSS. We find that the GNSS‐only inversion and GNSS and GRACE joint inversion show larger long‐term ∆TWS than the GRACE solutions and hydrological models across the Sierra Nevada Mountains, Klamath Mountains, Coastal Ranges, and the Central Valley, particularly during the second drought period. In addition, the inversion results show larger annual fluctuations of TWS in the northern mountain ranges than GRACE. Many studies have found good agreement between GRACE and GNSS annual vertical amplitudes where water loading signals are large and regionally persistent (e.g., Fu & Freymueller, 2012; Tregoning et al., 2009). However, other studies have pointed out that terrestrial water loading may only make up a portion of the annual loading deformation signal and that GNSS and GRACE may not agree where the seasonal hydrologic loading signal is small compared to other seasonal signals or noise in the GNSS time series (e.g., Chanard et al., 2020; Jiang et al., 2013; Li et al., 2016; Ray et al., 2008). In California, where tectonic deformation likely overprints the vertical GNSS signal, combining GRACE with GNSS helps to limit the effect nonhydrologic loading has on long‐term ∆TWS estimates. This can be seen most clearly in the residual GNSS vertical velocity (Figure 7a), which shows the largest residuals in places where vertical tectonic signals are also present. In other tectonically active regions, the long‐term agreement between GRACE and GNSS is weak, but has been used to correct GNSS stations for hydrologic loading in order to isolate the tectonic component (e.g., Fu & Freymueller, 2012). The residual vertical velocity likely principally contains tectonic deformation and so can be similarly used.

Previous studies in California have found disagreement between GNSS‐derived ∆TWS estimates, GRACE‐derived ∆TWS estimates, and predicted ∆TWS from hydrological models. For example, Argus et al. (2014) found disagreement between GRACE and GNSS‐derived annual TWS amplitudes. Using GNSS time series from 1994 to 2013, Argus et al. (2014) find an annual amplitude of up to 0.6 m‐equivalent water height in the Sierra Nevada Mountains, which is almost twice as large as the annual amplitude found using our joint inversion framework and approximately four times as large as the GRACE solutions. However, the resolution of their result is significantly better than GRACE and the annual amplitude of ∆TWS quickly goes to ∼0–0.1 m in the adjacent Basin and Range while GRACE remains at ∼0.15 m‐equivalent water height. Despite the application of leakage error corrections, the GRACE JPL mascon solutions may still contain leakage between mascon areas with high and low TWS fluctuations. Studies have shown that mascon solutions cannot capture ∆TWS over basins smaller than ∼100,000 km^2^ (e.g., Zhang et al., 2019). This may be why the GNSS inversion results show larger mass change over the mountain ranges and lower mass change over the Basin and Range than the GRACE solutions. Improved GNSS and GRACE joint inversions can be accomplished with additional leakage corrections for smaller basins applied to the GRACE solutions. Similarly, the use of rescaling to reduce leakage between physiographic provinces from the implementation of Laplacian smoothing in the inversion may further improve the result (e.g., Enzminger et al., 2018).

In addition, Enzminger et al. (2019) found that GNSS‐derived annual water storage changes were an order of magnitude larger than estimates from the North American Land Data Assimilation System (NLDAS‐2) Noah Land Surface Model (LSM) and Variable Infiltration Capacity (VIC) model in the Sierra Nevada Mountains. Their findings confirm the supposition put forth in Argus et al. (2017) that Noah LSM's do not capture the complete TWS variations that may exist in the Sierra Nevada Mountains (Enzminger et al., 2019). We also find significant differences between our inversion results and the WGHM and GLDAS hydrological models in both annual ∆TWS (as in Enziminger et al., 2019) and multiyear ∆TWS (as in Argus et al., 2017) over the Sierra Nevada Mountains and the northern mountain ranges, extending the findings by both authors that complete ∆TWS may not be captured by these hydrological models.

## 6 Conclusions

This study presents a new framework to combine GNSS vertical deformation time series and GRACE data products to solve for spatiotemporally variable ∆TWS. Key concepts of our approach include the following:

1. Joint inversion of GNSS vertical deformation and GRACE ∆TWS to combine the complementary spatiotemporal sensitivity to ∆TWS of each observation system
2. Continuous wavelet transform‐based approach to correct steps in and denoise GNSS time series
3. Wavelet decomposition of input signals in order to preserve spectral frequency content of the ∆TWS signals while performing a joint inversion
4. Determining the optimal weighting between GNSS and GRACE by finding a balance between model roughness and model misfit and considering known long‐term tectonic deformation that is superimposed on GNSS time series

We use this inversion method to solve for monthly ∆TWS across California and Western Nevada from January 2003 to December 2016. The joint inversion method used here allows us to achieve increased spatial resolution compared to GRACE while utilizing GRACE's unique ability to provide regional closure of the water budget. Using a continuous wavelet transform, we preserve spectral contents, including subannual, annual, and multiyear components of the ∆TWS signal, without assuming a functional form for the temporal behavior of the signal. We show that results from our GNSS and GRACE mascon joint inversion are regionally in agreement with GRACE CSR mascon solutions and GRACE COST‐G solutions. However, our results show improved isolation of larger magnitude signals over regions where we expect large ∆TWS signals (mountain ranges and the Central Valley Aquifer) and smaller magnitude signals over regions where we expect smaller ∆TWS (Basin and Range). In addition, we show that, in general, our ∆TWS estimates are larger than those from the WGHM and GLDAS hydrological models, indicating that these models may not capture long‐term ∆TWS or water stored deeper than the shallow subsurface.

The presented approach is directly transferrable to any region worldwide where a sufficient density of GNSS sites exists. In addition, the approach is also applicable for a joint inversion of GRACE data with elastic loading deformation maps based on InSAR analysis, where the elastic loading signal is present and not overprinted by other geophysical deformation signals. Given the increasing availability of SAR acquisitions since the launch of Sentinel‐1 in 2014 and the upcoming launch of NISAR, our approach will be useful for estimating ∆TWS maps with a high spatial resolution for most regions around the world.

The resulting ∆TWS maps are applicable for hydrological studies such as drought monitoring or water balance analysis and water management approaches that require water storage changes at higher spatial resolution than the GRACE observations alone provide. Also, geophysical studies that need to correct for the effects of water storage changes in signals or isolate the tectonic component in GNSS observations can benefit from the presented approach. Our results for the joint inversion of GNSS and GRACE are available for download in the form of spatiotemporal maps of ∆TWS from January 2003‐December 2016 across California and Western Nevada at the link provided in the acknowledgments.

## Supporting information

Supporting Information S1

## Data Availability

GRACE JPL mascon data products are downloaded from https://podaac.jpl.nasa.gov/dataset/TELLUS_GRACGRFO_MASCON_CRI_GRID_RL06_V2. GNSS time series are downloaded from the Nevada Geodetic Laboratory (http://geodesy.unr.edu/gps_timeseries/tenv3/IGS14/). GRACE CSR mascon products are downloaded from http://www2.csr.utexas.edu/grace/RL06_mascons.html. GRACE COST‐G solutions are downloaded from ftp://isdcftp.gfz-potsdam.de/grace/GravIS/COST-G/Level-3/TWS. Data from the GLDASCLSM hydrological model is downloaded from https://disc.gsfc.nasa.gov/datasets/GLDAS_CLSM10_M_2.1/_summary?keywords=GLDAS%20v2.1. Hannes Müller Schmied (hannes.mueller.schmied@em.uni-frankfurt.de) at the University of Frankfurt provided global ∆TWS maps from the WGHM model version 2.2d. Corrections applied to the GNSS timeseries are from W.R. Peltier at the University of Toronto (https://www.atmosp.physics.utoronto.ca/~peltier/data.php) for the GIA model ICE6G_D and from the Earth‐System‐Modelling Group at GFZ for the NTAL (http://esmdata.gfz-potsdam.de:8080/repository/entry/show?entryid=80daee1b-ff73-481f-b0f3-18026282c03e) and NTOL (http://esmdata.gfz-potsdam.de:8080/repository/entry/show?entryid=94df5183-aec2-41b5-ac14-e785a3e30c15) corrections.
