# Supplementary material for: Joint Inversion of GNSS and GRACE for Terrestrial Water Storage Change in California
Source: J Geophys Res Solid Earth. 2022 Mar 25;127(3):e2021JB023135. doi: 10.1029/2021JB023135 (PMC9287077; doi:10.1029/2021JB023135)
Supplement: Supplementary file 1 — Supporting Information S1 [file JGRB-127-0-s001.docx]

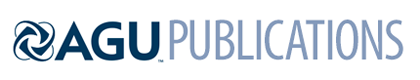


*Journal of Geophysical Research: Solid Earth*

Supporting Information for

**Joint Inversion of GNSS and GRACE for Terrestrial Water Storage Change in California**

G. Carlson,^1*^ S. Werth^1^, M. Shirzaei^1^

^1^ Department of Geological Sciences, Virginia Polytechnic and State University, Blacksburg, VA, USA

**Contents of this file:**

Text S1-S3

Figures S1 – S12

**Additional Supporting Information (files uploaded separately):**

Contained in this document is:

1. A discussion on the bound setting procedure (Text S1, Figure S1 and S2)
2. Description of the Kalman filter applied and additional timeseries without the Kalman filter applied (Text S2, Figure S3)
3. Description of a common step-correction method and comparison with our CWT-based step correction method (Text S3, Figure S4 and S5)
4. Fit to coseismic step and postseismic deformation from the El Mayor Cucupah Earthquake (Figure S6)
5. Locations of stations removed that show dominant poroelastic deformation, volcanic deformation, and sediment compaction (Figure S7)
6. Vertical velocity of GIA (ICE6G_D) (Figure S8)
7. Additional timeseries ∆TWS averaged over JPL mascon regions and physiographic provinces (Figure S9)
8. Standard deviation of inversion results (Figure S10)
9. Weighted root-mean-square error (Figure S11)
10. Correlation between GNSS and GRACE (Figure S12)

**Text S1. Bound Setting**

We set bounds for the inversion of the long-term component of ∆TWS using the long-term rate of ∆TWS during 5 different epochs: pre-drought (Jan 2003-Dec 2006), first drought (Jan. 2007-Jan. 2010), inter-drought (Feb 2010-Sept 2011), second drought (Oct. 2011- Sept. 2015), and post-drought (Oct 2015-Dec 2016). We first remove the short-term component of each GNSS and GRACE timeseries using the continuous wavelet transform- based signal decomposition (Equations 1-3 and Section 3.3 in the main text). We then fit a linear trend to the remaining long-term component of each GNSS and GRACE timeseries over each epoch. Finally, we invert for the rate of TWS (rTWS) over each epoch at each grid cell. The lower (lb) and upper (ub) bounds for each time step for each grid cell beginning at time 1 (t1) and ending at time 2 (t2) are:

$$lb=rTWS*\left( t2-t1 \right)/365 - 0.05$$

$$ub=rTWS*\left( t2-t1 \right)/365 + 0.05.$$

Because we remove GNSS stations that are dominated by poroelastic deformation, we lose information over the Central Valley Aquifer, a region where significant ∆TWS occurs. In order to constrain water loss in the Central Valley, we use groundwater storage loss estimates to set the bounds of the inversion during both drought periods. We forward-model InSAR-derived groundwater loss estimates from Ojha et al. (2018) for the first drought period (Jan. 2007 – Jan. 2010) and Ojha et al. (2019) for the second drought period (Oct. 2011 – Sept. 2015) to solve for elastic vertical deformation on an evenly spaced grid of 0.1 degrees over the Central Valley. In order to limit oversampling of the Central Valley region as compared to other regions within our study area, we only maintain grid points that show elastic uplift larger than 2 mm/yr. These observation points become “synthetic GNSS stations” and are included in the bound-setting inversions in the same way real GNSS stations are included. For the purposes of bound-setting, we choose the start and end date of the two drought periods based on the start and end date of the study periods in Ojha et al. (2018) and Ojha et al. (2019); though the start and end date of the first drought period is typically considered to be Oct. 2006-Oct. 2009. The “synthetic GNSS stations” are not included in the final long-term inversion and are only applied in order to set the bounds for the final long-term portion of the step-wise inversion. Inversion bounds for the short-term component of the inversion are +/- 0.3 meters per time step.


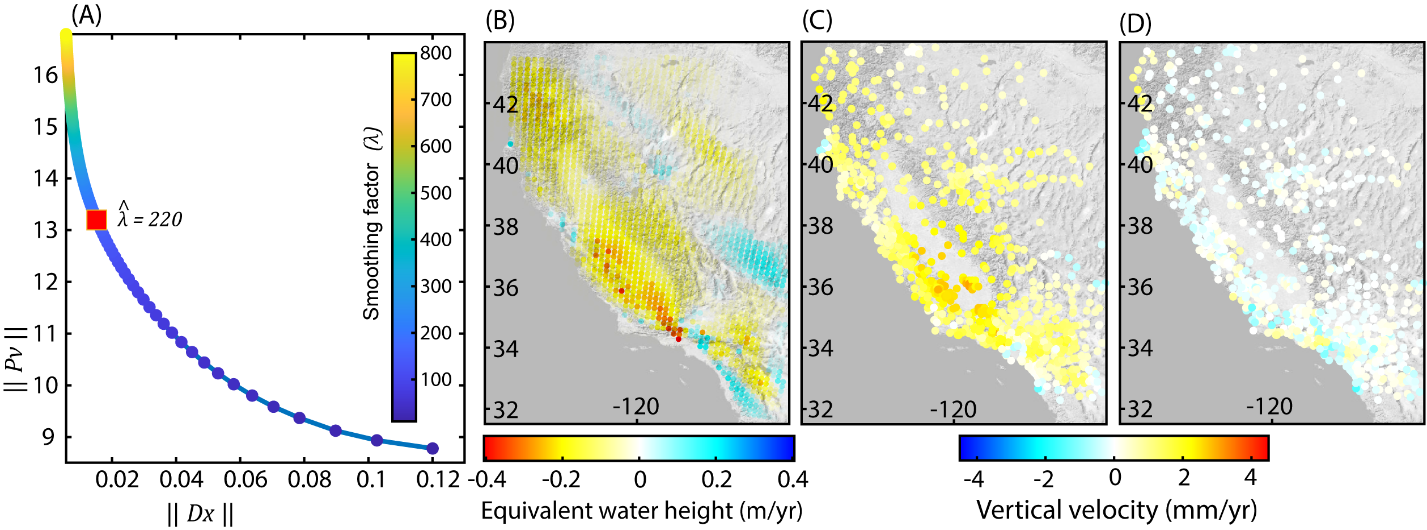


**Figure S1**. Example of inversion parameter selection over the epoch covering the second drought period (Oct. 2011 – Oct. 2015) for the GNSS-only inversion. **(A)** Trade-off curve. Red square indicates the optimal λ value ($\hat{\lambda} = 220$). **(B)** Inversion result for TWS rate (rTWS). **(C)** Vertical velocity of GNSS stations calculated during the second drought period. Observations in the Central Valley are “synthetic GNSS stations” calculated using InSAR-derived water storage change estimates from Ojha et al. (2019). **(C)** Residual GNSS velocity.


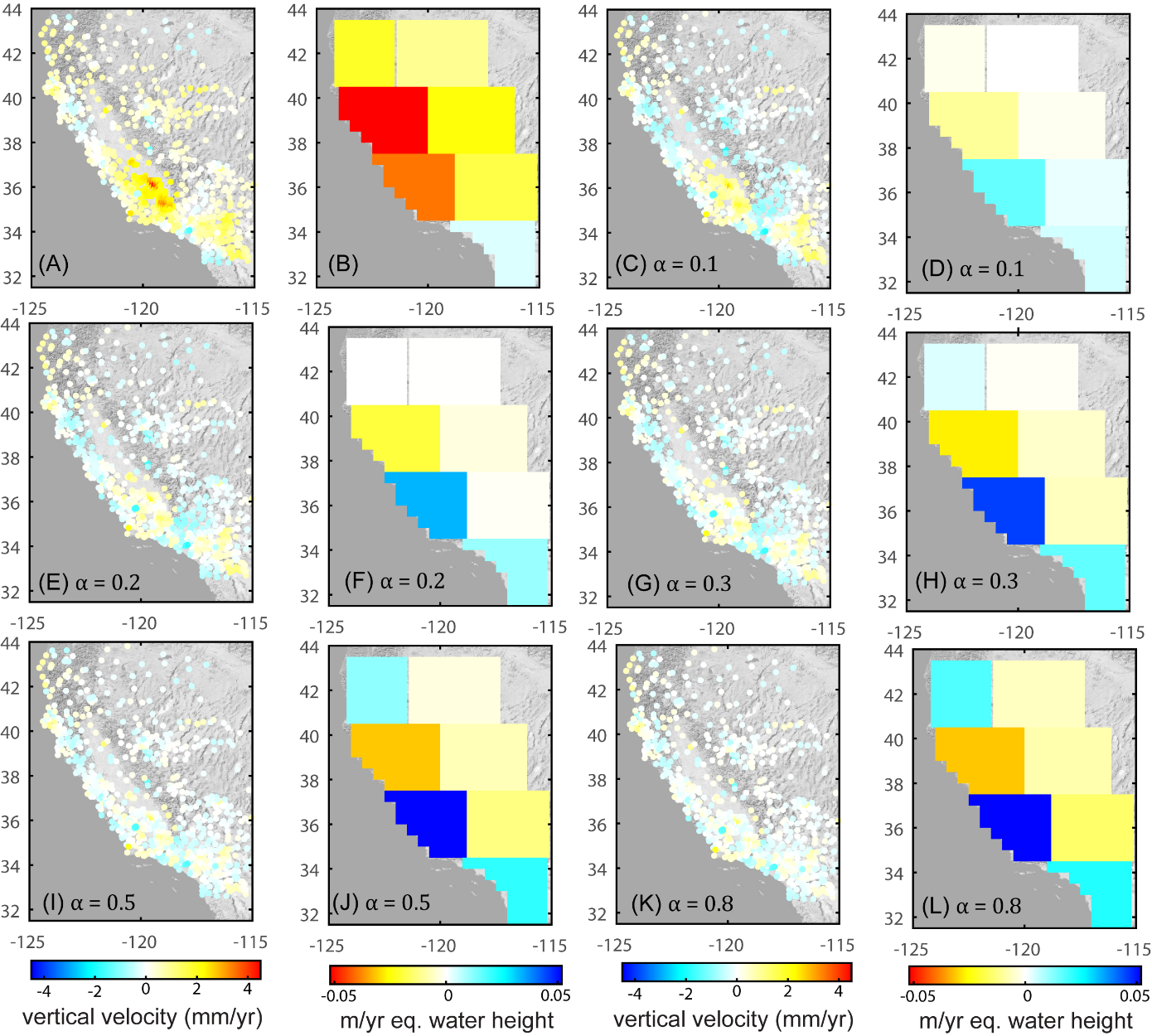


**Figure S2**. Example of inversion parameter selection over the epoch covering the first drought period (Jan. 2007 – Jan. 2010) for the GNSS and GRACE joint inversion. **(A)** vertical velocity of GNSS stations calculated during the first drought period. Observations in the Central Valley are “synthetic GNSS stations” calculated using InSAR-derived water storage change estimates from Ojha et al. (2018). **(B)** GRACE TWS rate for each mascon cell. **(C-L)** Residuals from the inversion results presented in Figure 4 in the main text for different values of α. (C, E, G, I, K) show residul vertical velocity. (D, F, H, J, L) show residual rTWS at the location of each mascon cell in m/yr.

**Text S2. Kalman Filter Framework**

Because each step in our inversion is considered independently, a more robust solution can be achieved by considering the whole timeseries as a dynamic system, whose state is perturbed by white noise. The linear Kalman filter can be used to estimate the optimal state of this dynamic system by using observations that are linearly related to the state of the system and statistical information summarized in the variance-covariance matrix of errors (Grewal and Andrews, 2001). Among many applications, Kalman filtering is commonly used to separate seasonal signal from noise in GNSS timeseries and identify time-varying trends and annual amplitudes (e.g. Didova et al., 2016; Klos et al., 2018). Here we used the recursive formulation of the linear Kalman filter implemented by Shirzaei et al. (2010).

Figure S3 shows a comparison between the Kalman filtered and original GNSS-only and joint inversion time series. The filter’s effects on the final time series are small, within the range determined by the variance-covariance matrices and the RMS difference between the filtered and non-filtered solutions are 0.020 meteres for the joint and 0.029 meters for the GNSS-only inversion.


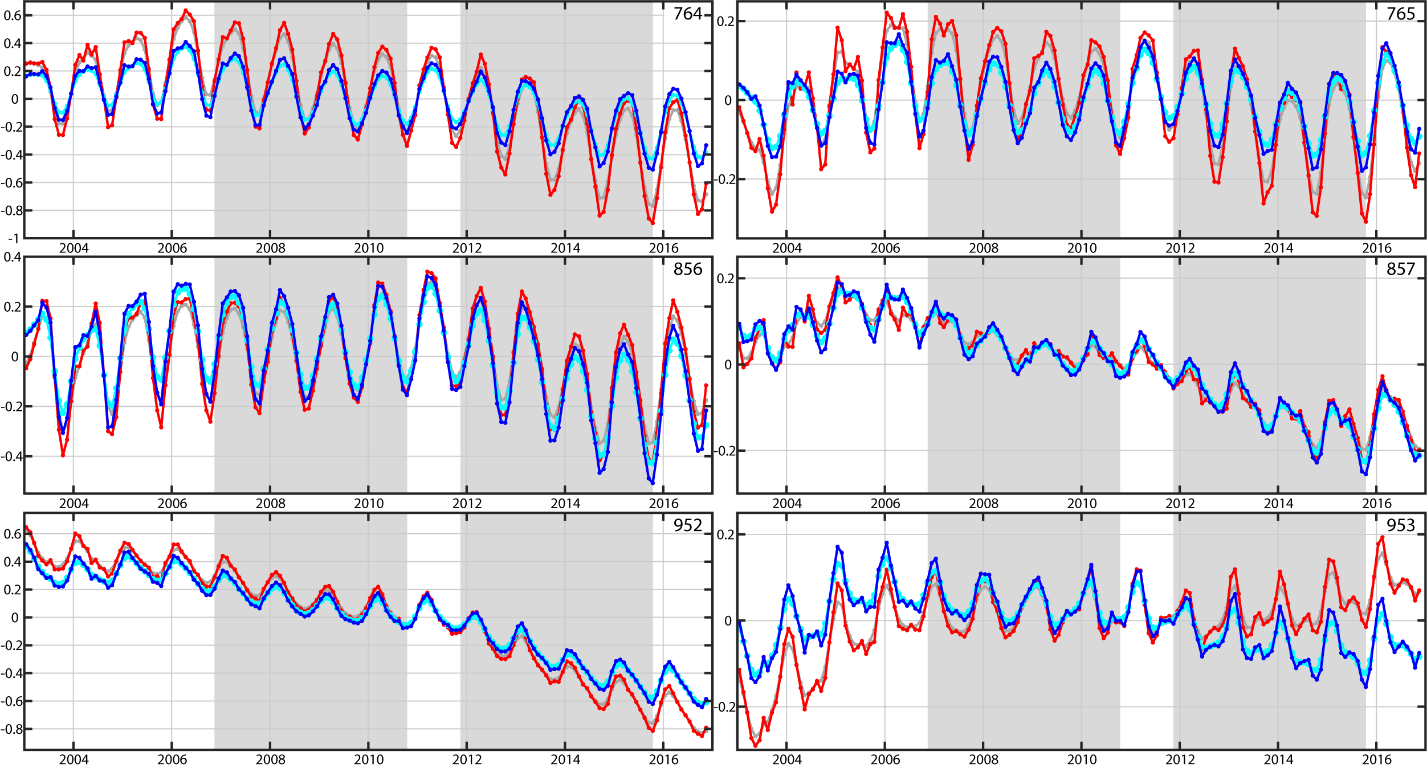


**Figure S3**. Comparison between the Kalman filtered solution (grey, cyan) and original solution (red, blue) for the GNSS-only inversion (grey, red) and joint inversion (cyan, blue) averaged over the mascon areas. Location of each mascon area can be found in Figure 1 and is identified by the numbers listed in the upper right of each panel.

**Text S3. GNSS timeseries step correction method**

GNSS timeseries ($x(t)$) can be fit with a functional model containing a constant offset ($x_{0}$), velocity ($v_{x}$), acceleration ($a_{x}$), and seasonal components for semi-annual, annual, and bi-annual periods as follows (e.g. Bevis and Brown, 2014):

$$x(t)=x_{0}+v_{x}(t-t_{o})+a_{x}{(t-t_{o})}^{2}+\sum_{k=1}^{3} [b_{k}sin( \omega_{k}(t-t_{o}))+c_{k}cos(\omega_{k}(t-t_{o}))]+\sum_{j=1}^{nj} s_{j}\mathcal{H}(t-t_{o})+\sum_{i=1}^{ni} d_{i}\log(1+\frac{\Delta t}{\tau})$$

Where $\omega_{k}$ are angular periods corresponding to periods of 0.5 years, 1 year, and 2 years. The last two terms are additional offsets due to steps fit with the Heaviside function ($\mathcal{H}$) and post-seismic deformation from earthquakes fit with a logarithmic function.

Though the step catalog contains documented offsets, additional offsets may be present and documented offsets may be undetectable in the vertical component. Correcting GNSS timeseries offsets is challenging and often offsets are not detected or falsely detected, resulting in mis-modeled components in the functional model. The wavelet-based step correction method described in section 2.1 in the main text is advantageous because no functional model needs to be estimated and fit to the timeseries, thus maintaining signal components that are not present in the functional model above. In order to compare the wavelet-based step correction method with a traditional step-correction method, we fit the timeseries with the functional model above using a non-linear least-squares fit. Steps are identified using the step catalog provided by the Nevada Geodetic Laboratory and additional steps that might be missing from the step catalog are identified using a threshold of 4 cm or temporal gaps larger than 60 days between observations. After fitting the functional model, we remove the steps and post-seismic deformation from the full timeseries. We then use a moving mean with a 14-day window centered on the observation of interest to smooth and reduce outliers. Comparison timeseries between this method and the CWT-based step-correction method is shown in Figure S4.


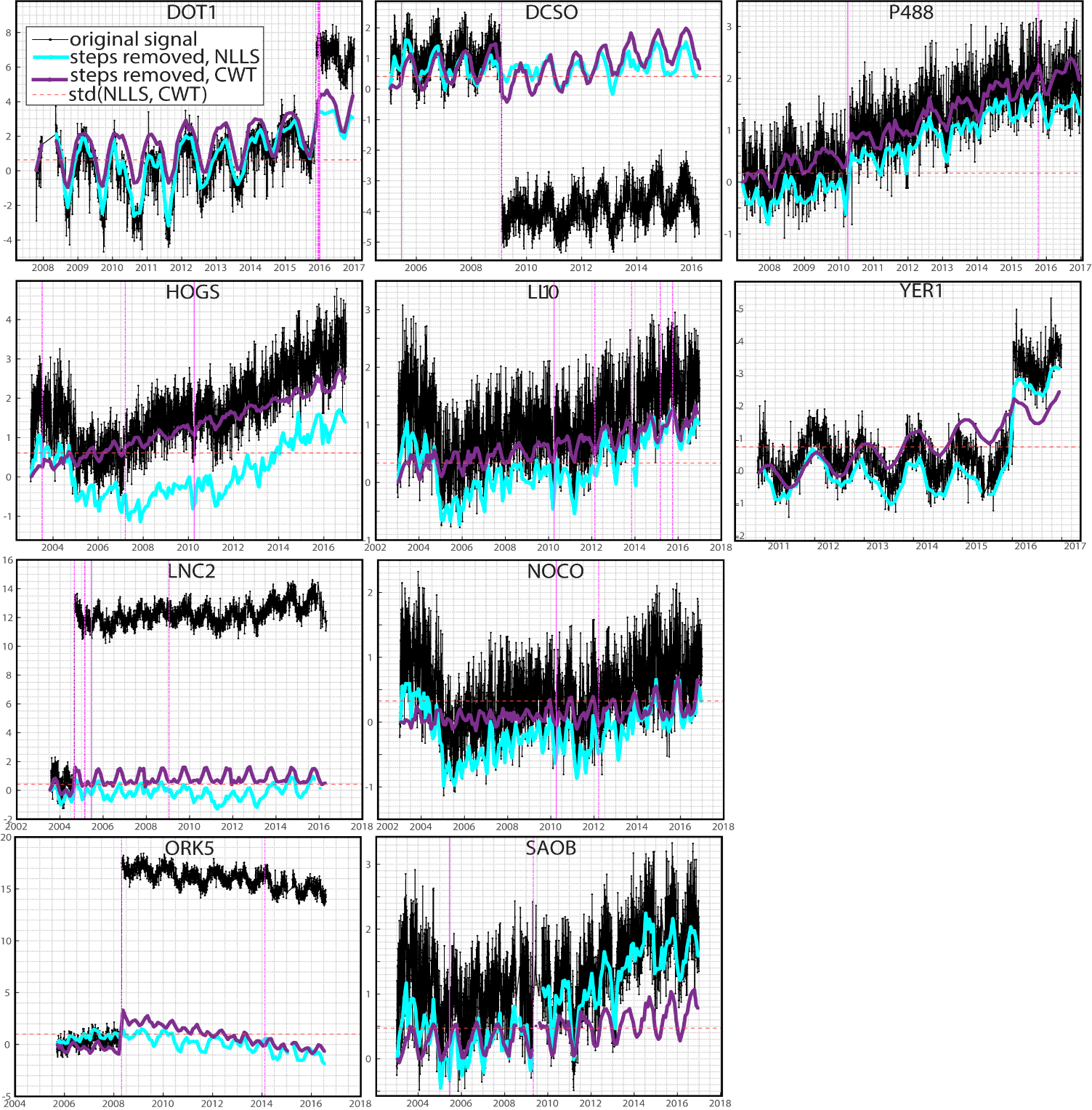


**Figure S4**. Timeseries comparing our CWT-based step correction method (cyan) with a commonly-used step correction method (blue) for GNSS stations (names are listed above each panel). All timeseries are given in cm of vertical displacement. Vertical magenta lines show steps and dashed red line shows standard deviation between the two timeseries using the two different correction methods. A constant initial value is removed from each timeseries such that the beginning of the timeseries is set to zero.

In addition, we perform a synthetic test by generating 5000 timeseries using the functional model above where the coefficients $v_{x}$, $a_{x}$, $A_{j}$, $t_{j}$, and the amplitudes of the annual and semi-annual, and bi-annual components are randomly assigned based on values from our real GNSS timeseries. Random noise is added to each timeseries. We use both methods to correct the step and calculate the difference between the known timeseries with the step removed and the timeseries generated by removing the step using each method. We then calculate the difference between the mean value of the residuals 7 days before and 7 days after the step to estimate how well the step is corrected by each method. If the difference between the residuals on either side of the step is less than the standard deviation of the added noise, then we consider the step “corrected”. We run the test once for the case that the time of the step is known and fed into the functional model and one where the time of the step is unknown, as is the case for steps in the timeseries that are not recorded in the step catlog. Using the nonlinear least-squares fit to the functional model, the step is corrected 99% when the time of the step is known. However, in the case where the time of the step is not known, the step is only corrected 31% of the time. This indicates that the non-linear least squares fitting approach that we are using here is reliant on the a priori information on the timing of the steps. On the other hand, our wavelet-based step correction method is successful ~90% of the time in both cases when the timing of the step is known and when it is not known. When considering the real GNSS timeseries, we estimate that ~1/5 of the steps are identified using our threshold of 4 cm between observations or a gap of more than 60 days. However, these values are somewhat arbitrary and so some steps not listed in the step catalog may go undetected. In addition, the nonlinear least squares step correction method has an advantage over the wavelet based step correction method for this synthetic test because we are generating a timeseries using a functional model and fitting the timeseries with the same functional model. Thus, considering the variability in real GNSS timeseries and considering that many steps are not found in the step catalog, we determine that the wavelet-based step correction method works similarly as well as a conventional nonlinear least squares fit to a functional model.


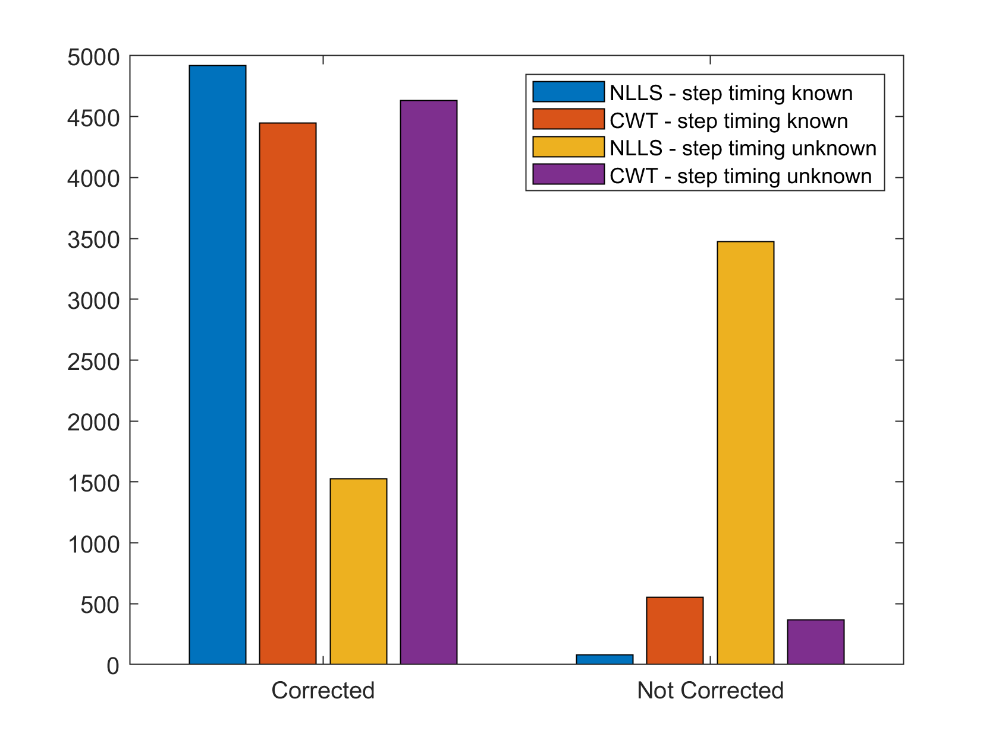


**Figure S5**. Synthetic test results. NLLS = Non-linear least squares step correction method and CWT = continuous wavelet transform- based step correction method.


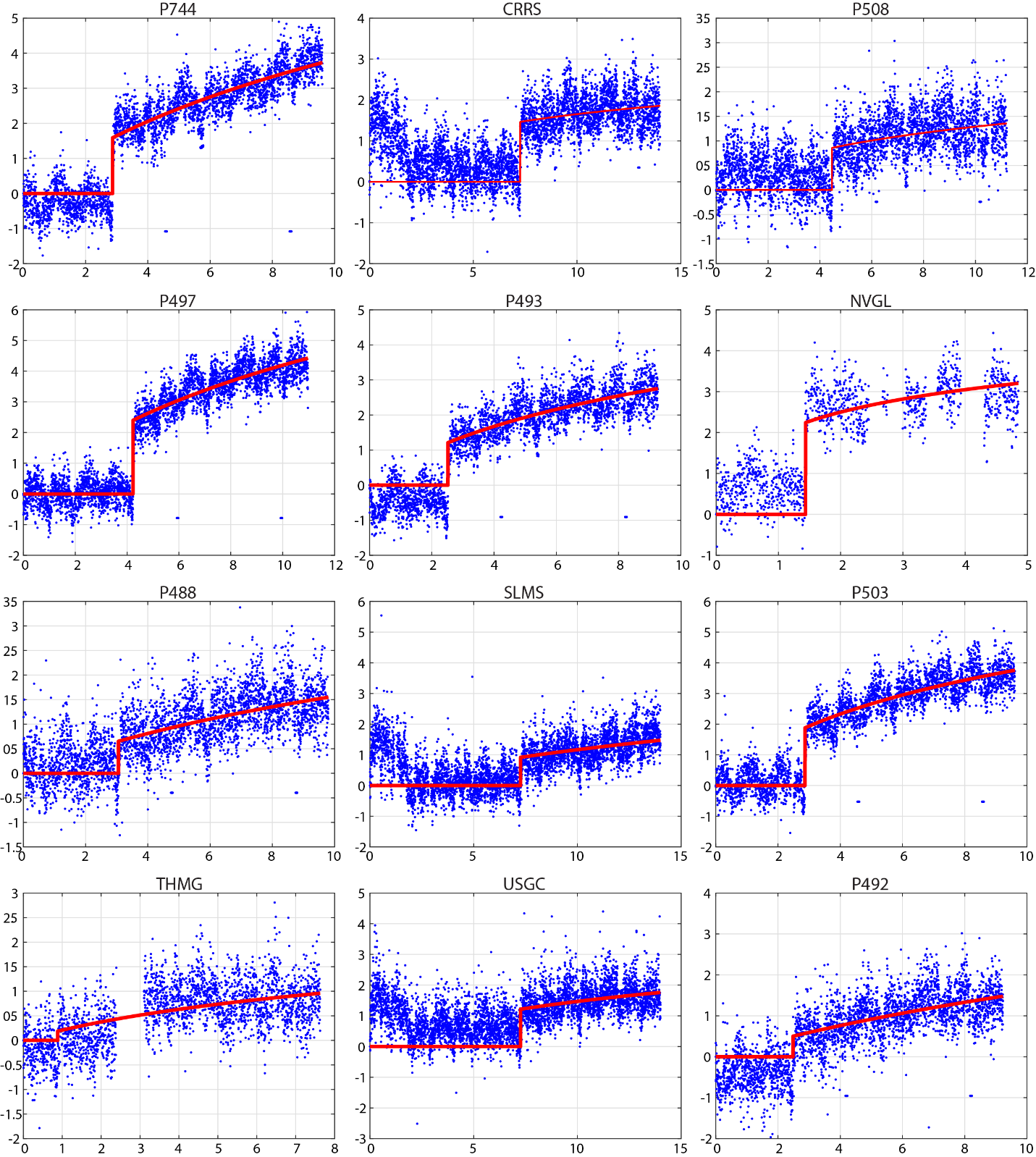


**Figure S6**. Coseismic step and postseismic deformation fit to timeseries impacted by the El Mayor Cucupah earthquake for 12 GNSS stations (names are listed above each panel). X-axis shows time in years since the start of the timeseries, Y-axis shows displacement in centimeters.


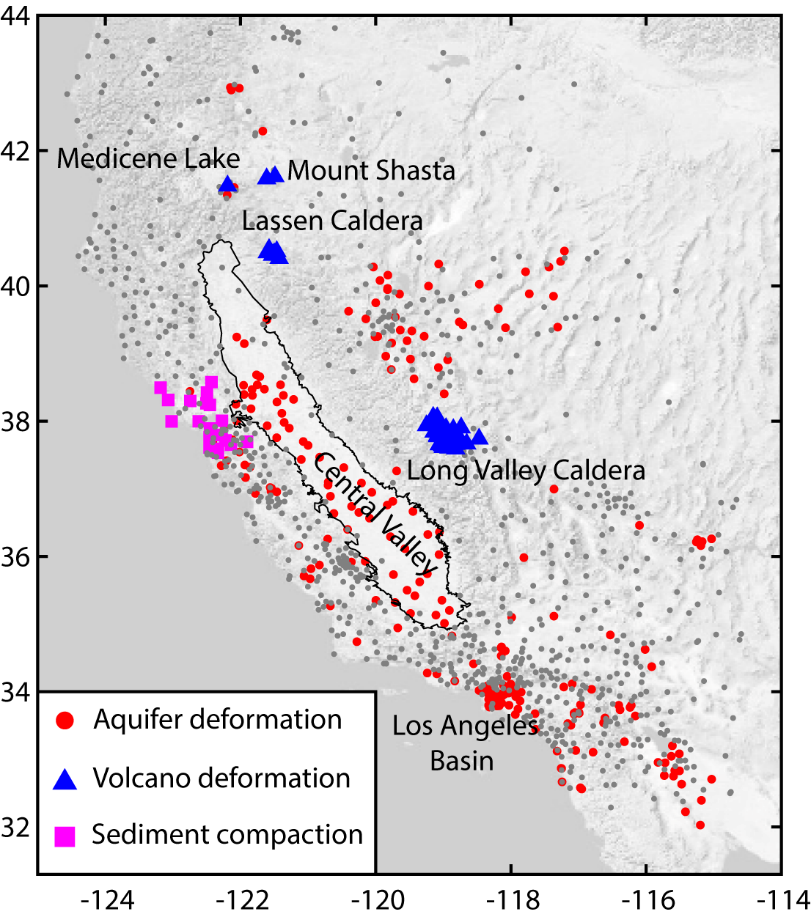


**Figure S7**. Stations that are not considered in the inversion are removed because their dominant deformation signal originates from volcanic deformation (blue trienagles), aquifer deformation (red circles), or sediment compaction (purple squares). Grey points show locations of GNSS stations that are considered in the inversion. Volcanic regions and major aquifer-systems are labelled.

**
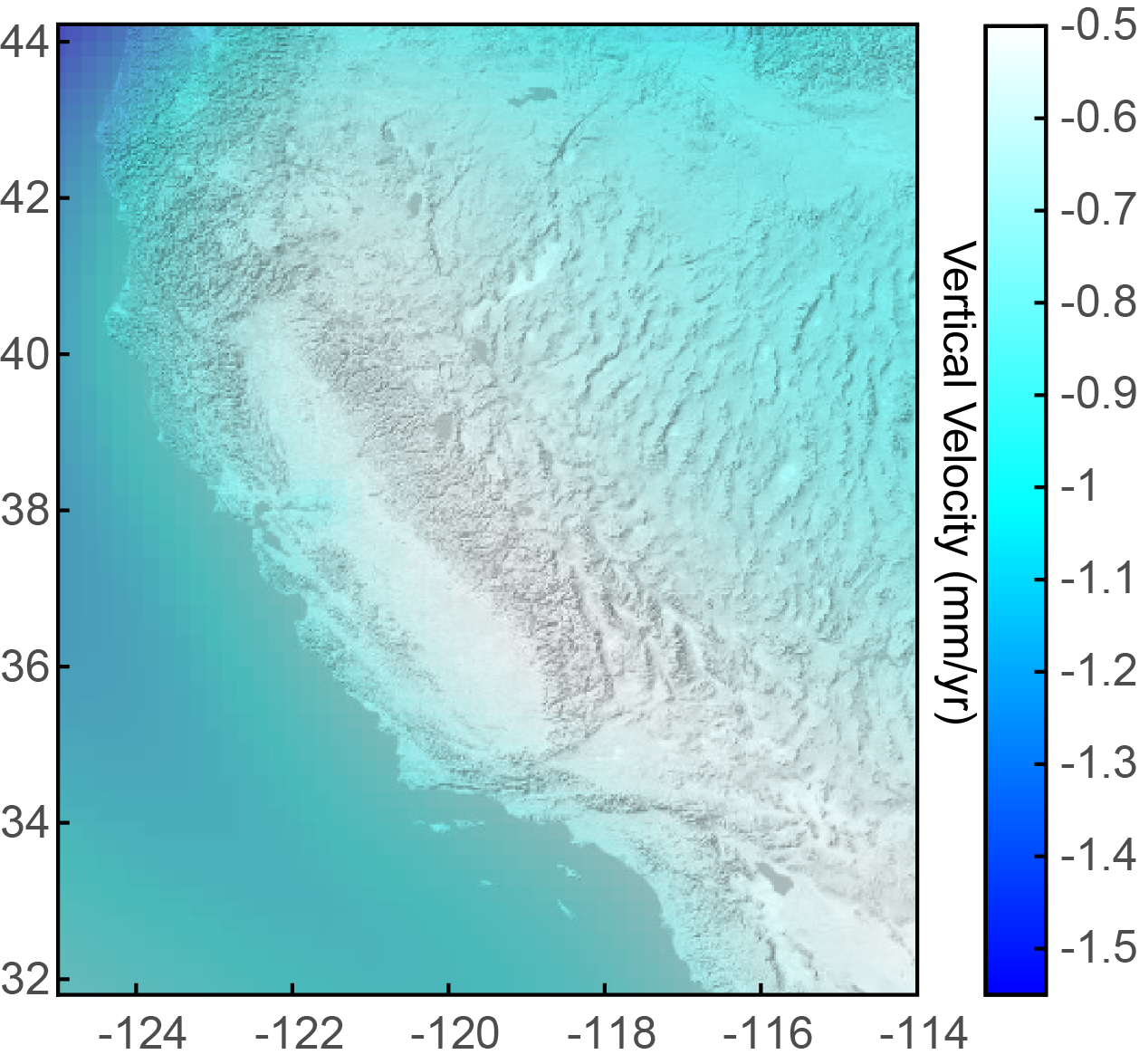
**

**Figure S8**. Long-term vertical velocity of Glacial Isostatic Adjustment from ICE6G_D.


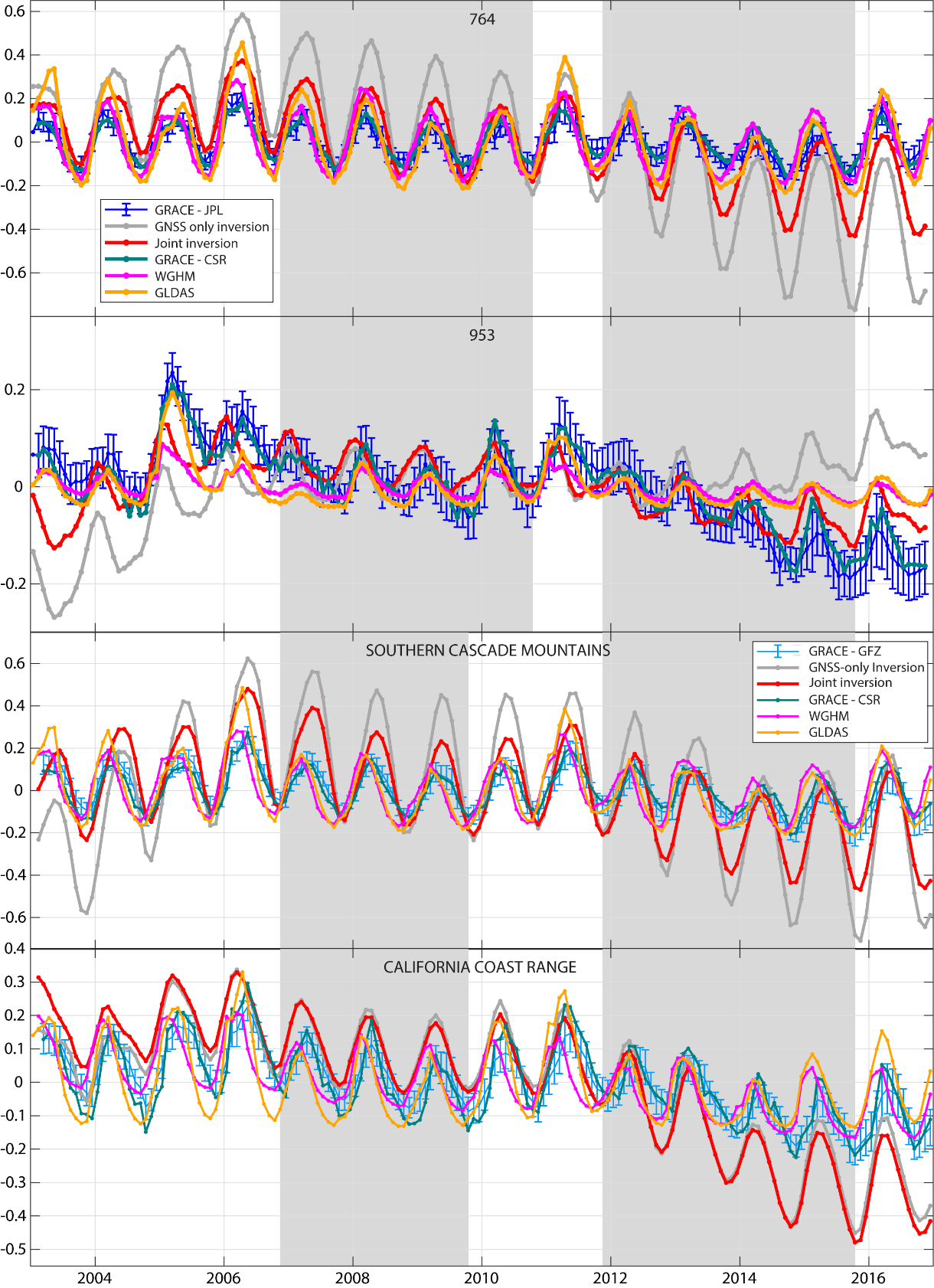


**Figure S9**. Timeseries of ∆TWS averaged over JPL mascon regions (panels 1 and 2) and over the remaining physiographicprovinces (panels 3 and 4). All timeseries are given in m-equivalent water height and zeroed at the mean value.


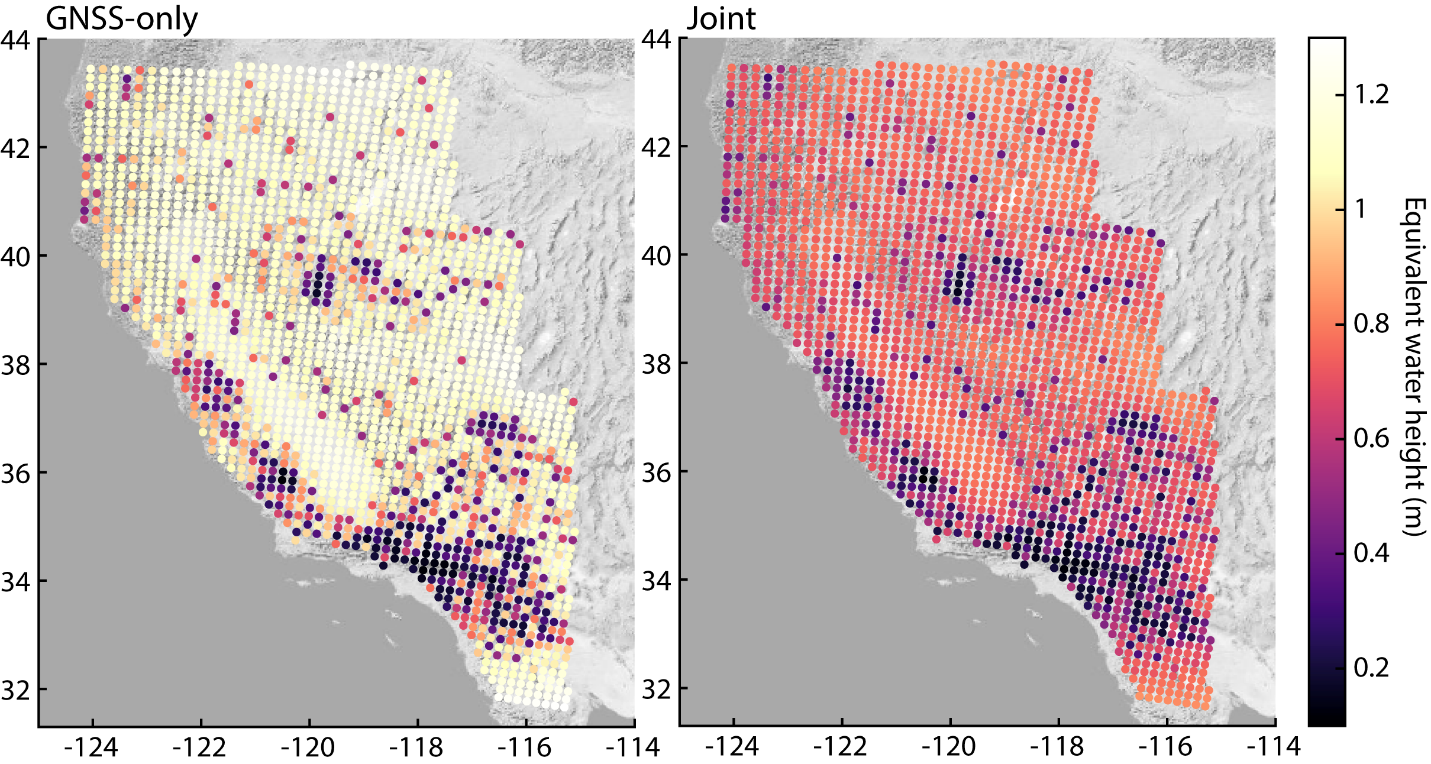


**Figure S10**. Mean standard error using equation 11 in the main text of the GNSS-only inversion (left panel) and GNSS and GRACE joint inversion result (right panel) in m-equivalent water height. Mean standard error is 0.97 for the GNSS-only inversion and 0.66 for the joint inversion.


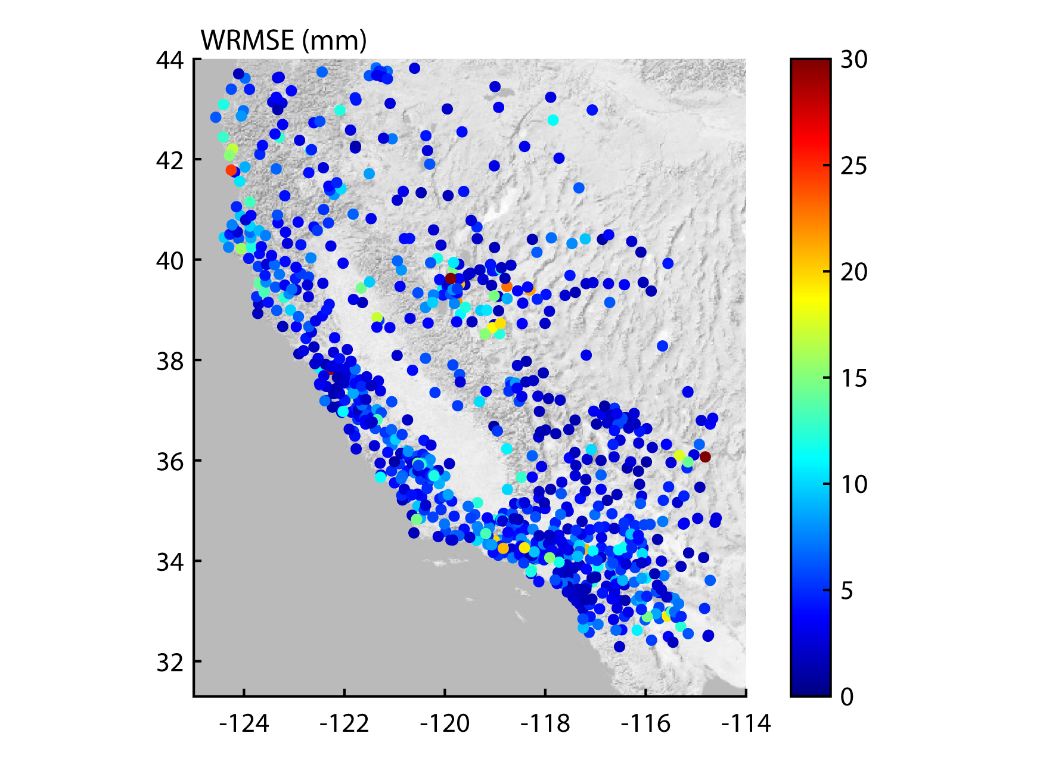


**Figure S11**. Weighted root mean square error (WRMSE) between the forward-modelled vertical deformation from the GNSS and GRACE joint inversion calculated at GNSS station locations compared to observed GNSS observations.


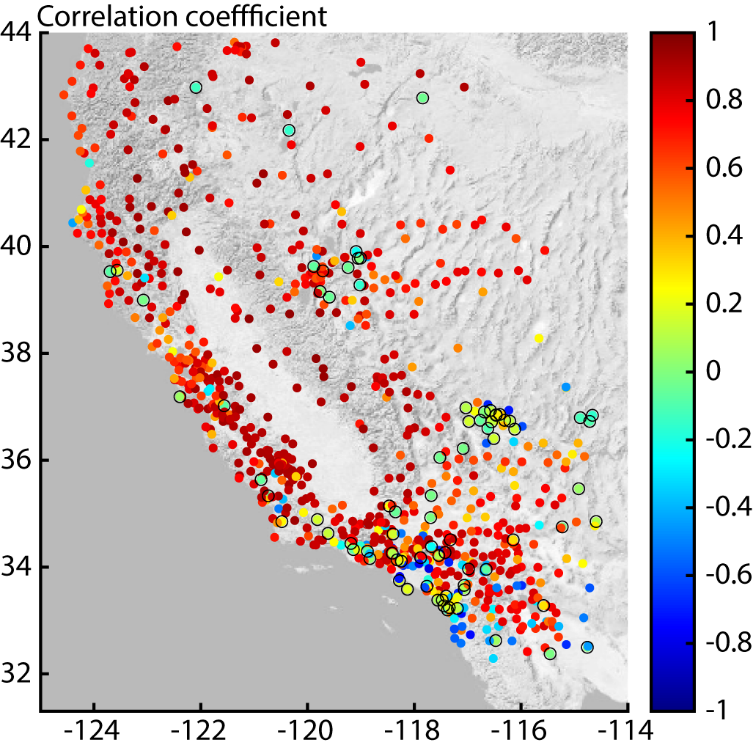


**Figure S12**. Correlation coefficient between GNSS and GRACE timeseries. GRACE JPL mascon data products are used to forward-calculate deformation at the location of GNSS stations. Black outlines show correlation coefficients not considered significant at the 95% confidence level (~10% of stations). Correlation between GNSS and GRACE is high over most of the study area. Some stations are anti-correlated with GRACE, especially in southern California.

**Citations**

Bevis, M., & Brown, A. (2014), Trajectory models and reference frames for crustal

motion Geodesy, Journal of Geodesy, 88(3), 283-311, doi: 10.1007/s00190-013-0685-5.

Didova, O., Gunter, B., Riva, R. et al. (2016), An approach for estimating time-variable rates

from geodetic time series, J Geod 90, 1207–1221.

https://doi.org/10.1007/s00190-016-0918-5

Grewal, M. S., and A. P. Andrews (2001), *Kalman Filtering : Theory and Practice Using MATLAB*, 416 pp., Wiley-Interscience.

Klos, A., Bos, M.S. & Bogusz, J. (2018), Detecting time-varying seasonal signal in GPS

position time series with different noise levels, GPS Solut 22, 21. https://doi.org/10.1007/s10291-017-0686-6

Ojha, C., M. Shirzaei, S. Werth, D.F. Argus, & T.G. Farr (2018), Sustained Groundwater loss in California's Central Valley exacerbated by intense drought periods. *Water Resour. Res.* **54**(7). 4449-4460 doi:10.1029/2017WR022250.

Ojha, C., Werth, S., & Shirzaei, M. (2019), Groundwater loss and aquifer system compaction in San Joaquin Valley during 2012–2015 drought, Journal of Geophysical Research: Solid Earth, 124, 3127– 3143. <https://doi.org/10.1029/2018JB016083>.

Shirzaei, M., and T. R. Walter (2010), Time-dependent volcano source monitoring using interferometric synthetic aperture radar time series: A combined genetic algorithm and Kalman filter approach, Journal of Geophysical Research-Solid Earth, 115, doi:Doi 10.1029/2010jb007476.
